# Population and sub-national (district) level diversity in missed and dropout of different doses of hepatitis-B vaccine among Indian children aged 12–59 months

**DOI:** 10.1371/journal.pgph.0000243

**Published:** 2022-05-17

**Authors:** Junaid Khan, Apurba Shil, Parul Puri

**Affiliations:** 1 LASI, International Institute for Population Sciences, Mumbai, Maharashtra, India; 2 Dept. of Public Health, Faculty of Health Sciences, Ben-Gurion University of the Negev, Beersheba, Israel; 3 International Institute for Population Sciences, Mumbai, Maharashtra, India; Universidad Nacional de Colombia, COLOMBIA

## Abstract

Initiating with a birth dose and a full immunization against hepatitis-B is crucial during early childhood in a country like India where maternal screening of hepatitis-B surface antigen is almost negligible and there is a considerable risk of vertical transmission among children. It is also evident that coverage of hepatitis-B is lowest among all other vaccine doses included in the universal immunization program. In addition, the major challenge is posed by the missed and drop-out of different doses of hepatitis-B among Indian children. In this context, this study examined the population and sub-national level diversity in missed and dropout of different doses of hepatitis-B vaccine in India. We analysed a large dataset of 196,654 children aged 12–59 months from a nationally representative cross-sectional survey, the National Family Health Survey (NFHS), 2015–16. Bivariate cross tabulation was used to estimate the prevalence and the dropout rates. Multivariable-adjusted logistic regression was applied to assess the likelihood of the study events. Within a Bayesian framework, a district-level spatial analysis was conducted employing the Besag-York-Mollie (BYM) Model and the Leroux Model. During 2016, 38% of the children missed the birth dose nationally and 45% of the children did not complete full immunization of hepatitis-B. Findings suggest, presence of socio-economic and demographic gradients in missed and drop-out of different doses of hepatitis-B at national level. The sub-national level spatial analysis identifies more than 280 (out of 640) districts with substantially higher risk (Posterior Median Risk>1) in terms of missed and drop-out of different doses. Most of these districts are scattered across the North-Eastern and Northern part of India. The findings hint the existence of a population and sub-national level diversity in India’s missed out and dropout of hepatitis-B doses. Identifying high risk population sub-groups and the districts with children at higher risk of missing the birth and consecutive doses informs the existing knowledge base and helps in formulating community-oriented policies and programs.

## Introduction

To address the issues that matter most to the community and the planet, United Nations (UN) started three years of vigorous community consultation at the 2012 UN Conference on Sustainable Development in Rio de Janeiro [1]. With a global agenda of leaving no one behind, 17 global goals and 169 targets were introduced under the umbrella of the Sustainable Development Goals (SDG) in 2015. In view of achieving these goals holistically, the GAVI Alliance (formerly known as the Global Alliance for Vaccines and Immunization), recommended Immunization as one of the “best buys in global health” [2]. The agency further explained that immunization holds a central position in achieving 14 of the 17 goals proposed by the United Nations (UN) [2].

The burden of Hepatitis B infection is not same throughout the World. Therefore, to identify the problem prone areas, all the countries were classified into three sub-groups, on the basis of their endemicity, namely high (8% or more), intermediate (2–7%), and low endemicity (less than 2%) [3, 4]. The south-east Asia, including India, belong to the intermediate to high endemicity region [5]. Furthermore, owing to the large population size, India holds second position in the burden of chronic hepatitis-B infection in the World [6].

India introduced their first ever immunization program, Expanded Programme of Immunization (EPI) in the year in 1977–78, however this program was limited in its reach and was catering exclusively to the needs of the urban population [7]. A nationwide program, Universal Immunization Programme (UIP) was introduced in 1985, which included five vaccines, namely Bacillus Calmette-Guérin (BCG), oral polio vaccine (OPV), Diphtheria, Pertussis, Tetanus (DPT), typhoid-paratyphoid vaccines and measles. However, it was only in the year 2007 that UIP included hepatitis-B vaccination [5]. Presently, the Universal Immunization Programme (UIP) includes nine Vaccine Preventable Diseases (VPDs), namely diphtheria, pertussis, tetanus, polio, tuberculosis, measles, hepatitis B, japanese encephalitis, and meningitis and pneumonia caused by the haemophilus influenza type B [8].

WHO estimates suggests that 80–90 percent of infants and 30–50 percent of the children under six years of age develop chronic infections in the later stages of the life if they do not receive HBV as scheduled [9]. The recent estimates generated by the National Family Health Survey (NFHS), 2015–16 suggests that despite the inclusion of HBV in the UIP for more than a decade, the coverage of hepatitis-B vaccination is lowest among all the other vaccinations included in the program [10]. The estimates suggest that in 2016, around 34 percent of the children failed to receive the birth dose of hepatitis-B vaccination [10]. National Immunization Schedule (NIS) proposes a total of four doses of hepatitis-B vaccination, including a birth dose (to be given within 24 hours of the birth), primary dose one (to be given in the sixth week of birth), primary dose two (to be given in the tenth week of birth), and primary dose three (to be given in the fourteenth week of birth) [10]. According to the National Immunization Schedule (NIS), the timings of the first, second and third dosage of HBV coincides with that of DPT vaccine [8]. However, the estimates generated by NFHS-4 suggest that the drop-out rates between first to second (f2s) and second to third (s2t) doses is higher for HBV (dropout rates: f2s: 7.2% and s3t: 24.18%) than DPT vaccination (dropout rates: f2s: 3.8% and s3t: 7.3%) [10].

In spite of the aforementioned issues, till date there are no studies which provide insights on the patterns and potential predictors of missed birth dose and drop-out rates after first and second doses of HBV in India. Hence, the present study aims to examine the pattern of missing the birth dose and drop-out to the consecutive doses of HBV among children aged 12–59 months across various states and Union Territories (UTs) of India. Additionally, the study identifies the demographic and socio-economic differentials of missed birth dose and drop-out rates after first and second doses of hepatitis-B vaccination in India [10].

## Methods

### Study design and participants

National Family Health Survey (NFHS) is a cross-sectional household survey conducted across India during January 20, 2015 and December 4, 2016. India is the second most populous country in the world after China. A share of 9% of children under age five contribute to the total population count [11]. The primary administrative units are states in India. These states are then sub-divided into districts which are the second administrative units. According to Census 2011, there are 640 districts spread over 36 states and union territories. The NFHS, 2015–16 survey used the Census 2011 sampling frame to select the enumeration areas. Census enumeration blocks in urban areas and villages in rural areas constitute the sampling frame of primary sampling units (PSUs) and PSUs were selected using probability proportional to size (PPS) method. The details of the survey design, instruments and reports are available in public [10].

Children aged 12–59 months across the country are the study population. And a total of 196,654 children aged 12–59 months were utilized to conduct the unit level as well as the district level analysis.

The fourth round of the National Family Health Survey, 2015–16 used a stratified, two-stage cluster sampling design using the enumeration areas (EAs) of the 2011 India census as the primary sampling unit (PSU) and households as the secondary sampling unit (SSU). In the first stage, 645 census enumeration blocks in urban areas and villages in rural areas were selected with probability proportional to EAs size (PPS) from the complete list of EAs created for the sampling frame. In the second stage of selection, a fixed number of 22 households per cluster were selected with an equal probability systematic selection from the newly created household listing. Finally, 601,509 households were successfully interviewed, yielding a response rate of 98%. A total of 259,627 children aged 0–59 months constitute the surveyed sample population of the children. The detailed methodology has been published in the 2015–16 NFHS final report [10].

The 2015–16 National Family Health Survey (NFHS) data for India is publicly available and could be downloaded upon data request from the Measure DHS program website at https://www.dhsprogram.com/data/dataset_admin. Following the definition of NFHS, the study variables were defined and were analysed.

The district level shape file of India was retrieved from GitHub through https://github.com/datameet/maps/ [12], which was shared under Creative Commons Attribution 4.0 license. Then it was re-projected in WGS 1984 UTM zone. Due to unavailability of survey data for parts of Jammu and Kashmir and Gujarat (Ran), these areas are illustrated as missing in the generated maps.

### Measures

Demographic Health Surveys (DHS) provide child immunization estimates considering children aged 12–23 months. Also, this age group has been traditionally used to provide vaccination coverage estimates. Despite this, DHS collects information on all the doses for all the children under age five. To mention NFHS, like all other demographic health surveys, also records the source of information on childhood vaccination. A detailed description of the vaccination reporting by each dose of hepatitis-B is provided in the **S1 Table.**

As per the NFHS-4, there are 48,928 children in the age range of 12–23 months. Thus, if the analysis is restricted to children aged 12–23 months, information on 147,726 children aged 24–59 months will be missed, introducing a sampling bias in the estimation. Moreover, considering the age group of 12–59 months covers a diverse group of children born during 2010–2015. Additionally, as the study employs a district-level analysis, including all children aged 12–59 months helps with good sample size.

Moreover, the World Health Organizations’ (WHO) reference manual suggests that the target population of children is usually 12–23 months if the final primary vaccination is at nine months of age. In another case, the targeted population could be 24–35 months for the vaccine doses like Measles-containing-vaccine second-dose (MCV2) and diphtheria, pertussis, and tetanus-containing vaccine four (DTPCV4). For other supplementary immunization activities, the age group could be less than five years or higher. Thus, there is no stringent criterion to select a target population while studying the vaccination coverage [13, 14].

*Mission Indradhanush* was launched in 2014, is an immunization coverage program that targets all children up to five years in the poorly performing districts across India. Thus, children under age five should be studied to understand immunization coverage better. This study would be a benchmark to provide an exhaustive understanding of missed and drop out of different hepatitis-B doses. This study attempts to estimate the coverage using a comprehensive approach to understand the broader picture of vaccination coverage among under-five children in India.

For the birth dose, including the successive doses, we assumed the children to be vaccinated if they were found to be vaccinated in the vaccination card or from the mother’s self-reporting. The “don’t know” cases (constituting 1.8 percent of the total cases) reported by the mothers were not considered as vaccinated.

The study included seven outcome variables, missed birth dose (hepatitis-B0), missed first dose (hepatitis-B1), missed second dose (hepatitis-B2), missed third dose (hepatitis-B3), first dose dropout, second dose dropout and third dose dropout.

### Operational definitions

**Missed birth dose (hepatitis-B0):** children (aged 12–59 months) who did not receive the hepatitis-B vaccine dose within 24 hours of birth.

**Missed first dose (hepatitis-B1)**: children (aged 12–59 months) who did not receive the first dose at the 6^th^ week of delivery.

**Missed second dose (hepatitis-B2):** children (aged 12–59 months) who did not receive the second dose at the 10^th^ week of delivery.

**Missed third dose (hepatitis-B3):** children (aged 12–59 months) who did not receive the third dose at the 14^th^ week of delivery.

**First dose dropout:** Children (aged 12–59 months) who received the birth dose but missed the first dose are the dropouts between birth & first dose.

**Second dose dropout:** Children (aged 12–59 months) who received the first dose but missed the second dose are considered to be the dropouts between first & second dose.

**Third dose dropout:** Children (aged 12–59 months) who received the second dose but missed the third dose are considered to be the dropouts between second & third dose.

With an exhaustive literature review, the study included a set of variables related to child, mother and household characteristics as potential predictors. **S2 Table** provides a description of the potential risk factors along with their categories. It is worth mentioning that multicollinearity was tested before proceeding with the statistical analysis. Conventionally, variance inflation factor (VIF) helps to detect the multicollinearity in regression analysis. In particular, the variance inflation factor for the estimated regression coefficient b_j_, denoted by VIF_j_ is the value by which the variance of b_j_ is inflated by the existence of correlation among the predictor variables in the model.

The variance inflation factor for the *j*^*th*^ predictor is:

$$VIF_{j}=\frac{1}{1-R_{j}^{2}}$$

Where,

$R_{j}^{2}$ is the value of R^2^ obtained by regressing the j^th^ predictor on the remaining predictors

As the variables selected in this study are all categorical variables, the normal VIF does not hold true as R^2^ does not exist in case of a categorical dependent variable. Taking care of the nature of the variable, we employed the generalized variance-inflation factor (GVIF) analysis following the definition of Fox and Monette, 1992 [15]. **S3 Table** presents the values of generalized variance-inflation factors (GVIF) as well as the adjusted generalized variance-inflation factors (AGVIF) for each of the models being estimated in this study. According to the usual rule of thumb, values between five and ten indicate moderate multicollinearity and values above ten indicate high multicollinearity with the predictors. The estimation was executed using the CAR package (version 3.0–12) in the R version 4.1.0.

### Statistical analysis

#### Unit level analysi

To compute the prevalence of missing a dose, we considered the total sample population of the children (aged 12–59 months) as the denominator. Thus, the prevalence of missing a particular dose is defined as follows:

$$Prevalence\;(missing\;the\;i^{th}\;dose)=\frac{Number\;of\;children\;missed\;the\;i-th\;dose}{Total\;sampled\;children\;aged\;12–59\;months}$$

for i = 0,1,2,3

To calculate the drop-out rate between two successive doses of hepatitis-B (i, i+1), we considered the children who received the i^th^ dose in the denominator and those who missed the (i+1)^th^ dose in the numerator. And thus, the dropout rate for a particular dose is defined as follows:

$$Dropout\;rate\;for\;{\left(i+1\right)}^{th}\;dose=\frac{Number\;of\;children\;who\;received\;the\;i-th\;dose\;but\;not\;received\;the\;i+1\;th\;dose}{Number\;of\;children\;who\;received\;the\;i-th\;dose});$$

for i = 0, 1, 2,

Bivariate analysis was used to estimate the prevalence and dropout rates of different doses of hepatitis-B vaccine by the set of variables which defined a child’s socio-economic and demographic characteristics. Both the prevalence and dropout rate were expressed in terms of per 100 children. Multivariable logistic regression estimation was done to examine the odds of missing/dropping out a dose.

Furthermore, the study presented population attributable risk (PAR) to quantify the contribution of the socio-demographic factors on the different doses of hepatitis B vaccination drop outs. In the literature, PAR estimates the excess rate of disease, mortality or any such events in the total study population of exposed and unexposed individuals that is attributable to the exposure [16–18]. In the present case, PAR shows the proportion of different vaccination drop out that can be attributed to the independent variables. The PAR estimation mainly relies on the relative risk estimates generated for individual predictor from the fitted multivariable logistic regression models. The PAR calculation is based on comparing the “observed scenarios” with “idealistic scenarios” [19]. This measure basically demonstrates that an extent of different dose of hepatitis B vaccination dropouts would be reduced if the “observed scenarios” of the predictors will be replaced by the “idealistic scenarios” in the population. The formula to estimate PAR is as follows:

$$PAR=\frac{(RR-1)*P}{(RR-1)*P+1}$$

Where, RR is the relative risk of each predictor on the outcomes (it is the adjusted odds ratio generated from the fitted multivariable logistic regression models for each outcome variables) and P is the prevalence of the predictor of interest. We generated all the PAR estimates in the study by utilizing the ‘*regpar’* package in STATA (version 14.0) [19]. The package also provides standard errors, z–statistics, P–values and 95% confidence intervals (CIs) for each PAR estimates. To increase the interpretability, we multiplied 100 with each PAR estimates being generated.

#### District level spatial analysis (Bayesian mapping)

To examine the pattern of sub-national (districts) level risk estimates of missing birth dose, first dose, second dose, third dose and dropout of different doses a novel district level analysis has been carried out. To do so, we first computed the district level Standardized Incidence Ratio (SIR) to measure the risk associated with each of the outcome variables. For district i (i = 1, 2, 3…,640), the SIR of an outcome variable was measured as the ratio between observed (Y_i_) and expected (E_i_) counts of that outcome variable:

$${SIR}_{i}=\frac{Y_{i}}{E_{i}}$$

The value of SIR_i_ for an outcome variable informed whether the i^th^ district had risk greater than ‘1’ (SIR_i_ > 1), equal to ‘1’ (SIR_i_ = 1) or lesser than ‘1’ (SIR_i_ < 1) comparing the cases for that event against the expected from the standard population. Moreover, the expected counts (E_i_) of an outcome variable depicted the total number of children missing a particular dose or being dropped out of a dose that one would expect if the population of the district behaved the same way like the standard population. In this study, latest district level age ((in months): 12–23, 24–35, 36–47, 48–59)-sex (male, female) stratified Census 2011 data of India [20] was utilized as the standard population to compute the expected counts across the districts as described elsewhere [21].

At the next step, an array of statistical models (including both frequentist and Bayesian approach) was fitted to extract the model based smoothed risk of the events under study in order to identify the districts that carried a significantly higher risk of the prevalence. Initially, we fitted a generalized Poisson’ regression model for each of the outcome variables and then we checked the existence of spatial autocorrelation on the residuals of the model by Moran’s *I* statistic and its significance [22]. A statistically significant spatial autocorrelation of the model residuals leads to [23] inclusion of spatial regression models in the study. Taking care of the spatial autocorrelation in the residuals of the Poisson model, we fitted a series of Conditional Autoregressive (CAR) Models within a Bayesian framework including Besag-York-Mollie (BYM) Model [23] and the Leroux Model [24].

We included selected district level covariates as the potential risk factors associated with the missing and drop out of different doses of hepatitis-B across districts. These variables were selected as per the strength of relationship with the outcome variables at the individual level analysis. The inferences were drawn based on Markov Chain Monte-Carlo (MCMC) simulation which helped to combine the prior distribution with the data and a final estimation of the posterior likelihood [25]. The Deviance Information Criteria (DIC) was used to compare the goodness of fit of the models fitted. The model with the lowest DIC was selected. We further checked the convergence of the fitted Markov Chains by drawing the trace plots of the samples for each of the model parameters [25]. Additionally, Gelman-Rubin statistics were computed for the convergence check where a value less than 1.1 indicated the existence of convergence. All the Markov Chains were run for several thousand “burn-in” iterations to achieve convergence.

Further we computed the Posterior Median Risk (PMR) and Posterior Exceedance Probability (PEP) from the fitted models. PMR is the ratio between fitted values and expected counts for each district which is basically the model based smoothed risk associated with the study events. Higher value of PMR (PMR>1) indicated the higher risk of the event. However, PEP was calculated using the district level posterior risk distribution which was the posterior probability that the PMR of vaccination drop out is greater than one given the data and PEP ranges from 0 to 1. Once we generated the district level SIR, PMR and PEP for each of the outcome variables, an array of interactive maps was generated to visualise the spatial distribution across 640 districts in India.

Stata 14.0 (STATA Corp LP, College Station, TX) was used to conduct the micro-level analysis. Whereas, R Studio1.3.1073 (2009–2019 RStudio, Inc) was employed to conduct macro-level analysis. Bayesian Spatial Modelling was performed using the R package “CARBAyes” version 5.2 [25]; and model convergence was assessed using the R package “Coda” version 0.19–4 [26]. The study followed the Strengthening the Reporting of Observational Studies in Epidemiology (STROBE) reporting guidelines for cross-sectional studies.

## Results

### Description of the children

**S4 Table** presents the distribution of children (aged 12–59 months) receiving different doses of hepatitis B vaccination. Nearly 40 percent of the children did not receive the birth dose of hepatitis B vaccine, 6 percent of the children dropped out after the first dose and 18 percent of the children were left out after the second dose.

**S5 Table** presents the distribution of the study sample (children aged 12–59 months) by their background characteristics. Almost one fourth of the study children belonged to 12–23 months of age group and mean age of the study population was 35 months. Thirty-seven percent of the children are of first birth order and 52 percent are male. Almost all the children were living with their mother and one third (32%) of the children’s mother had no formal education. Almost 40% of the children belong to either of the two socially excluded groups- Scheduled Castes/Tribes. The religious composition of the children shows that majority of (72%) of the children are Hindu followed by Muslim 16 percent and Christian 8 percent. Poverty situation prevails largely across India and half (50%) of the children belong to the poor wealth quintiles. Only 24 percent of the children belong from the urban part of and 26 percent of the children were born in home without any delivery care from skilled health personnel. The regional distribution of the children shows almost 30 percent of all the surveyed children came from Central part of India.

### Prevalence of missing different doses of hepatitis-B

For the first time NFHS collected information on different doses of hepatitis-B vaccination among under-five children. In India, almost two-fifth (38%) of the children did not receive the birth dose within 24 hours of birth, 21% of the children missed the first dose, 26% missed the second dose and 44% of the children missed the third dose (**Table 1**). The prevalence (per 100 children) of missing different doses of hepatitis-B varied substantially among the children from different sub-populations. Notably, the prevalence of missing the birth dose and third dose was significantly higher across all the sub-populations followed by second dose and first dose. Around 42% of the children aged 48–59 months did not receive the birth dose and 52% of the children in the same age missed the third dose. Children of higher birth order (4–5, 6+) showed critically low coverage of different doses of hepatitis-B (**Table 1**). More than three-fifth (61%) of the children of 6+ birth order did not receive the birth dose and the third dose; 41% of them missed the first dose and 46% of them missed the second dose. Result showed no male-female difference in the prevalence of missing different doses of hepatitis-B. A significant difference was observed in the coverage of different doses of hepatitis-B among the children by their mother’s educational attainment and children of mother with no education carried the burden of highest prevalence of missing the birth dose (49%) and for the rest of doses too than the rest of the children. By social caste, children from the scheduled tribe and scheduled castes carried the higher prevalence to miss different doses of hepatitis-B. A religion difference was also observed and the Muslim children showed the higher prevalence to miss the birth dose (47%) and the other respective doses. The coverage of different doses of hepatitis-B shows a wealth divide and children from the poorest quintile carry higher prevalence (50%) of missing the birth dose, the prevalence is 30% for the first dose, 35% for the second dose and the prevalence was 53% for the third dose. The rural-urban divide in the prevalence showed that children from the rural areas had comparatively low coverage of different doses of hepatitis-B than the urban children. **S6 Table** presents the prevalence of missing different doses of hepatitis-B by states/union territories in India.

**Table 1:** Prevalence (per 100 children) of missing different doses of hepatitis-B among children aged 12–59 months by background characteristics, National Family Health Survey (NFHS-4), India, 2015–16.

Background characteristics	Birth dose	First dose	Second dose	Third dose	Number of Children
**Child’s age (in months)**					
12–23	34.34	17.94	22.86	36.45	48,928
24–35	36.37	18.60	23.97	40.22	48,517
36–47	39.67	21.71	27.18	46.10	50,697
48–59	42.43	25.26	31.05	52.37	48,512
**Birth order**					
1	33.87	17.29	22.22	39.13	72,500
2_3	37.46	20.43	26.00	44.09	92,298
4–5	49.54	29.31	35.43	53.45	23,754
6+	61.41	40.66	46.26	61.41	8,102
**Sex of the child**					
Male	37.92	20.62	26.16	43.90	1,02,036
Female	38.52	21.15	26.37	43.66	94,618
**Mother’s education**					
No education	49.37	30.71	36.57	54.46	62,151
Primary or less	41.22	21.34	27.49	45.41	28,978
Secondary or less	32.70	16.14	21.15	38.05	87,696
Higher Education	25.37	12.07	16.65	35.29	17,829
**Social group**					
Scheduled Castes (SC)	37.74	19.73	25.10	42.19	36,789
Scheduled Tribes (ST)	41.19	25.07	30.84	48.63	39,356
Non-Scheduled Castes/Tribes	37.90	20.60	25.93	43.55	1,20,509
**Religion**					
Hindu	36.75	19.93	25.34	43.26	1,41,696
Muslim	47.41	26.83	32.55	49.29	31,026
Christian	36.89	20.34	26.06	42.77	16,101
Others	25.00	12.26	14.75	26.43	7,831
**Wealth quintiles** ^£^					
Poorest	50.26	29.54	35.20	52.53	51,477
Poorer	42.96	22.95	28.08	45.24	46,117
Middle	35.13	18.53	23.78	41.47	39,234
Richer	30.36	15.97	21.91	39.59	32,983
Richest	25.03	12.63	17.42	35.38	26,843
**Place of Residence**					
Urban	31.73	17.36	23.05	41.43	47,509
Rural	40.83	22.30	27.56	44.74	1,49,145
**Place of delivery**					
Home	61.05	34.25	39.83	55.77	49,278
Institutional	32.04	17.26	22.60	40.55	1,47,376
**Region**					
North	30.15	20.20	25.39	41.18	37,139
Central	47.98	26.12	32.60	51.99	55,902
East	43.52	18.19	21.50	35.83	40,969
North East	62.29	31.25	36.23	49.16	28,706
West	36.26	25.27	31.48	52.84	14,072
South	19.34	12.48	18.80	37.54	19,866
**Total**	**38.21**	**20.88**	**26.26**	**43.79**	**1,96,654**

£ wealth quintiles denote five different economic classes of India.

### Dropout rate of different doses of hepatitis-B

Dropout rates by children’s socio-economic and demographic characteristics are shown in **Table 2**. Children aged 12–59 months in India showed noticeable dropout rate in different doses of hepatitis-B and nationally, 6% of the children were dropped out between the birth dose and the first dose, 7% between the first dose to second dose and 24% between the second and the third dose (**Table 2**). The dose specific dropout rates were observed substantially different among the children from different sub-population. The dropout rate is significantly higher for the third dose than all other doses nationally and across all the sub-populations of children. Dropout rates were significantly higher among the children aged 48–59 months than all other children and the rates are 6%, 8% and 31% for the first, second and third dose, respectively. Among the children of 6+ ordered births, around 8% of them who received the birth dose were dropped out of the first dose and around 10% of the them who received the first dose were dropped out of the second dose whereas the dropout rate for the third dose was quite high (29%) among these children who received the second dose. Same as the missing prevalence, the dropout rates also did not show any large difference by gender of the children. Children of higher educated mothers showed comparatively lower rates of dropouts for each of the doses than the rest of the children of mother with less/no educated. Among the children of different caste categories, children from the Scheduled Tribe caste showed the higher dropout rates among them for different doses. Religion wise, the Muslim and the Christian children showed higher dropout rates than the rest of the children (Hindu and others). Children from the poorest wealth quintile carry higher dropout rates for each of the doses (first dose-7%; second dose-9% and third dose-27%) than those children from the richer wealth quintiles. There was almost no rural-urban difference observed in the dropout rates for different doses of hepatitis-B. Dropout rates for different doses of hepatitis-B were also observed lower among those children who were delivered in an institutional setting in the presence of a doctor or any skilled birth attendant. Among the different regions of India, Western region of India carry higher dropout rates for each of the doses. And the dropout rate is as high as 32% for the third dose among the second dose receiver in this part of India. **S7 Table** presents the prevalence of dropout rates of different doses of hepatitis-B by states/union territories in India.

**Table 2:** Dropout rates of different doses of hepatitis-B among children aged 12–59 months by background characteristics, National Family Health Survey (NFHS-4), India, 2015–16.

Background characteristics	First dose		Second dose		Third dose	
	Percentage	No of children^1^	Percentage	No of children^2^	Percentage	No of children^3^
**Child’s age (in months)**						
12–23	5.97	30,628	6.47	39,229	18.01	36,785
24–35	4.91	29,348	7.15	38,721	21.86	36,287
36–47	5.64	28,748	7.46	38,607	26.4	35,997
48–59	5.93	26,297	8.04	34,805	31.29	32,228
**Birth order**						
1	5.22	46,112	6.42	58,650	22.22	55,285
2_3	5.62	54,660	7.44	71,543	24.82	66,651
4–5	6.78	11,310	9.16	16,405	28.22	15,036
6+	8.19	2,939	9.92	4,764	28.67	4,325
**Sex of the child**						
Male	5.62	60,089	7.43	78,762	24.49	73,507
Female	5.6	54,932	7.08	72,600	23.85	67,790
**Mother’s education**						
No education	7.06	30,840	8.93	42,320	28.58	38,732
Primary or less	6.2	15,879	8.34	21,794	25.19	20,162
Secondary or less	5.05	55,460	6.41	71,567	21.88	67,451
Higher Education	4.3	12,842	5.63	15,681	22.68	14,952
**Social group**						
Scheduled Castes	5.62	22,668	7.14	29,082	23.2	27,128
Scheduled Tribes	6.54	18,816	8.1	27,537	26.06	25,498
Non-Scheduled Castes/Tribes	5.47	73,537	7.18	94,743	24.22	88,671
**Religion**						
Hindu	5.6	88,080	7.2	1,12,230	24.45	1,04,842
Muslim	6.19	16,474	8.27	22,280	25.14	20,596
Christian	5.72	5,374	7.82	10,383	22.76	9,627
Others	3.24	5,093	3.27	6,469	13.84	6,232
**Wealth quintiles** ^£^						
Poorest	6.67	25,213	8.55	35,510	27.24	32,590
Poorer	6.25	24,588	7.16	34,164	24.27	31,776
Middle	5.54	23,690	6.88	31,096	23.65	29,165
Richer	5.38	21,825	7.53	27,200	23.06	25,525
Richest	4.09	19,705	5.81	23,392	22.03	22,241
**Place of Residence**						
Urban	5.18	30,746	7.24	38,586	24.21	36,202
Rural	5.81	84,275	7.27	1,12,776	24.17	1,05,095
**Place of delivery**						
Home	6.51	16,891	9.03	30,766	26.83	28,017
Institutional	5.47	98,130	6.88	1,20,596	23.63	1,13,280
**Region**						
North	6.06	25,843	6.75	29,565	21.4	27,771
Central	6.53	30,527	9.3	41,920	29.28	38,319
East	4.22	24,594	4.53	33,551	18.68	32,218
North East	6.81	9,312	8.13	18,789	20.92	17,400
West	6.77	8,836	8.84	10,341	31.56	9,524
South	5.07	15,909	7.58	17,196	23.45	16,065
**Total**	**5.61**	**1,15,021**	**7.26**	**1,51,362**	**24.18**	**1,41,297**

£ wealth quintiles denote five different economic classes of India

^1^Children who received the birth dose

^2^Children who received the first dose

^3^Children who received the second dose

### Likelihood to miss different doses of hepatitis-B

The multivariable logistic regression estimation showed that child’s age, birth order, mother’s educational attainment, wealth status, place of delivery and region the child belong to were the significant predictors of missing different doses of hepatitis-B vaccination (**Table 3**).

**Table 3:** Adjusted odds ratio (AOR) of missing doses of hepatitis-B among children (12–59 months) by background characteristics, National Family Health Survey (NFHS-4), India, 2015–16.

Background characteristics	Missed Birth Dose	Missed First Dose	Missed Second Dose	Missed Third Dose
	AOR (95% CI)	AOR (95% CI)	AOR (95% CI)	AOR (95% CI)
**Child’s age (in months)**				
12–23 (Ref.)				
24–35	1.08***(1.04–1.12)	1.02(0.97–1.07)	1.04*(1.00–1.09)	1.16***(1.12–1.21)
36–47	1.22***(1.18–1.27)	1.21***(1.15–1.26)	1.21***(1.15–1.26)	1.46***(1.41–1.52)
48–59	1.33***(1.28–1.39)	1.44***(1.38–1.51)	1.43***(1.37–1.49)	1.87***(1.80–1.94)
**Birth order**				
1 (Ref.)				
2_3	0.98(0.95–1.01)	1.01(0.98–1.05)	1.03**(1.00–1.07)	1.08***(1.05–1.11)
4–5	1.08***(1.03–1.13)	1.14***(1.08–1.20)	1.16***(1.10–1.22)	1.20***(1.15–1.26)
6+	1.40***(1.30–1.50)	1.52***(1.42–1.64)	1.49***(1.39–1.61)	1.38***(1.29–1.49)
**Sex of the child**				
Male (Ref.)				
Female	1.01(0.98–1.04)	1.02(0.99–1.05)	1.00(0.97–1.03)	0.98(0.96–1.01)
**Mother’s education**				
Higher Education (Ref.)				
No education	1.29***(1.19–1.39)	1.66***(1.51–1.83)	1.66***(1.52–1.81)	1.46***(1.36–1.57)
Primary or less	1.10**(1.02–1.20)	1.17***(1.06–1.30)	1.23***(1.13–1.35)	1.11***(1.03–1.20)
Secondary or less	1.05(0.98–1.12)	1.07(0.98–1.17)	1.08*(0.99–1.17)	0.96(0.90–1.02)
**Social group**				
Non-Scheduled Castes/Tribes (Ref.)				
Scheduled Castes	0.98(0.94–1.03)	0.91***(0.86–0.96)	0.92***(0.88–0.97)	0.92***(0.88–0.96)
Scheduled Tribes	0.83***(0.78–0.89)	0.90***(0.84–0.97)	0.92**(0.86–0.99)	0.95*(0.89–1.01)
**Religion**				
Hindu (Ref.)				
Muslim	1.29***(1.22–1.37)	1.28***(1.21–1.37)	1.27***(1.20–1.35)	1.19***(1.13–1.27)
Christian	1.10(0.98–1.23)	1.14**(1.00–1.30)	1.13*(0.98–1.29)	1.10(0.97–1.24)
Others	0.63***(0.55–0.72)	0.59***(0.49–0.70)	0.53***(0.46–0.62)	0.49***(0.43–0.55)
**Wealth quintiles** ^**£**^				
Richest (Ref.)				
Poorest	1.54***(1.41–1.68)	2.08***(1.87–2.32)	2.01***(1.83–2.21)	1.86***(1.72–2.02)
Poorer	1.45***(1.34–1.57)	1.65***(1.49–1.83)	1.56***(1.43–1.71)	1.45***(1.34–1.56)
Middle	1.33***(1.23–1.43)	1.41***(1.28–1.55)	1.35***(1.24–1.47)	1.28***(1.19–1.37)
Richer	1.21***(1.12–1.30)	1.26***(1.14–1.38)	1.28***(1.17–1.39)	1.20***(1.12–1.29)
**Place of Residence**				
Urban (Ref.)				
Rural	0.98(0.92–1.04)	0.95(0.88–1.01)	0.93**(0.87–0.99)	0.91***(0.86–0.96)
**Place of delivery**				
Home (Ref.)				
Institutional	0.42***(0.41–0.44)	0.56***(0.54–0.58)	0.59***(0.57–0.61)	0.69***(0.67–0.72)
**Region**				
South (Ref.)				
North	1.58***(1.45–1.73)	1.48***(1.34–1.63)	1.26***(1.15–1.37)	1.06(0.99–1.15)
Central	2.69***(2.50–2.90)	1.49***(1.37–1.61)	1.31***(1.21–1.41)	1.25***(1.18–1.33)
East	2.11***(1.95–2.28)	0.84***(0.77–0.91)	0.66***(0.61–0.72)	0.59***(0.55–0.63)
North East	4.98***(4.55–5.45)	1.99***(1.80–2.19)	1.60***(1.46–1.75)	1.17***(1.09–1.26)
West	2.32***(2.10–2.56)	2.24***(2.01–2.50)	1.89***(1.72–2.09)	1.85***(1.70–2.01)

**Note**.

*p < 0.05

**p < 0.01

***p < 0.001.

Children aged 48–59 months (born during 2010–11) were 1.33 (AOR: 1.33, 95% CI: 1.28–1.39) times more likely to miss the birth dose than the children aged 12–23 months (**Table 3**). Similarly, these group of children were 1.44 (AOR: 1.33, 95% CI: 1.28–1.39) times more likely to miss the first dose, 1.43 (AOR: 1.33, 95% CI: 1.28–1.39) times more likely to miss the second dose and 1.87 (AOR: 1.33, 95% CI: 1.28–1.39) times more likely to miss the third dose.

The odds of missing different doses were quite high among children of higher 6+ birth order than the first ordered birth children. Children of 6+ birth order were 1.40 (AOR: 1.40, 95% CI: 1.30–1.50) times more likely to miss the birth dose, 1.52 (AOR: 1.52, 95% CI: 1.42–1.64) times more likely to miss the first dose, 1.49 (AOR: 1.49, 95% CI: 1.39–1.61) times more likely to miss the second dose and 1.38 (AOR: 1.38, 95% CI: 1.29–1.49) times more likely to miss the third dose than the children of first birth order. The odds of missing the birth dose were 1.29 (AOR: 1.29, 95% CI: 1.19–1.39) times more likely in children born to no educated mother and 1.1 (AOR: 1.10, 95% CI: 1.02–1.20) times more likely in children born to primary educated mothers than those children born to higher educated mother.

Children from the Scheduled Tribe class are 17% less likely to miss the birth dose, 10% less likely to miss the first dose, 8% less likely to miss the second dose and 5% less likely to miss the third dose than the reference group of children. The odds of missing the birth dose were 1.29 times higher among the Muslim children than the Hindu children and similarly the odds were higher for the other doses too.

The estimated adjusted odds ratio by wealth quintile showed a consistent decrease in odds across the higher wealth quintiles for each of the doses and compared the children from the richest wealth quintile; poorest wealth quintile children carried the higher odds to miss the different doses of hepatitis-B.

Children born in institutional facilities were always less likely to miss birth dose well as the other doses. Children born in institutional facilities were 58% (AOR: 0.42, 95% CI: 0.41–0.44) less likely to miss the birth dose than those children born in home (non-institutional facilities). Compared the Southern region, children from the North-East region of India were highly likely (AOR: 4.98, 95% CI: 4.55–5.45) to miss the birth dose followed by Central India (AOR: 2.69, 95% CI: 2.50–2.90) and western region of India (AOR: 2.32, 95% CI: 2.10–2.56).

### Likelihood to dropout of different doses of hepatitis-B

**Table 4** provides a detailed report from the multivariable logistic regression estimation of dropout of first, second and third dose of hepatitis-B. Children above age 24 months were consistently more likely to get dropped out of the second and the third dose and the odds were observed to be increasing with increasing age. The odds of third dose dropout were as high as 2.06 (AOR: 2.06, 95% CI: 1.96–2.18) among the children in the age group 48–59 months than those of age 12–23 months.

**Table 4:** Adjusted odds ratio (AOR) of dose dropout rates of hepatitis-B among children (12–59 months) by background characteristics, National Family Health Survey (NFHS-4), India, 2015–16.

Background characteristics	First Dose Dropout	Second Dose Dropout	Third Dose Dropout
	AOR (95% CI)	AOR (95% CI)	AOR (95% CI)
**Child’s age (in months)**			
12–23 (Ref.)			
24–35	0.81***(0.73–0.89)	1.11**(1.01–1.21)	1.28***(1.21–1.35)
36–47	0.93 (0.84–1.02)	1.14***(1.05–1.24)	1.63***(1.55–1.72)
48–59	0.96 (0.88–1.06)	1.22***(1.12–1.32)	2.06***(1.96–2.18)
**Birth order**			
1 (Ref.)			
2_3	1.00(0.93–1.08)	1.07**(1.00–1.15)	1.10***(1.06–1.14)
4–5	1.05(0.94–1.18)	1.17***(1.05–1.30)	1.16***(1.08–1.23)
6+	1.21**(1.00–1.46)	1.15*(1.00–1.33)	1.08(0.97–1.21)
**Sex of the child**			
Male (Ref.)			
Female	0.99(0.93–1.06)	0.95*(0.89–1.00)	0.97*(0.93–1.00)
**Mother’s education**			
Higher Education			
No education	1.32***(1.11–1.57)	1.45***(1.21–1.73)	1.19***(1.09–1.31)
Primary or less	1.17(0.97–1.41)	1.38***(1.16–1.64)	1.02(0.93–1.13)
Secondary or less	1.02(0.88–1.20)	1.09(0.92–1.29)	0.90***(0.83–0.97)
**Social group**			
Non-Scheduled Castes/Tribes (Ref.)			
Scheduled Castes	0.98(0.89–1.09)	0.99(0.91–1.07)	0.94**(0.89–0.99)
Scheduled Tribes	0.99(0.86–1.13)	0.99(0.87–1.12)	0.97(0.90–1.04)
**Religion**			
Hindu (Ref.)			
Muslim	1.10(0.98–1.24)	1.14**(1.03–1.27)	1.05(0.98–1.13)
Christian	1.12(0.83–1.51)	1.11(0.87–1.41)	1.02(0.87–1.21)
Others	0.57***(0.40–0.81)	0.47***(0.35–0.62)	0.51***(0.43–0.61)
**Wealth quintiles** ^**£**^			
Richest (Ref.)			
Poorest	1.76***(1.48–2.10)	1.55***(1.32–1.82)	1.47***(1.33–1.61)
Poorer	1.59***(1.35–1.87)	1.23***(1.06–1.43)	1.23***(1.12–1.35)
Middle	1.38***(1.17–1.62)	1.15*(0.99–1.33)	1.16***(1.06–1.27)
Richer	1.34***(1.15–1.57)	1.27***(1.08–1.51)	1.10**(1.01–1.20)
**Place of Residence**			
Urban (Ref.)			
Rural	0.96(0.86–1.08)	0.91*(0.83–1.00)	0.93**(0.87–1.00)
**Place of delivery**			
Home (Ref.)			
Institutional	0.94(0.85–1.03)	0.82***(0.76–0.87)	0.95*(0.91–1.00)
**Region**			
South (Ref.)			
North	1.24***(1.08–1.42)	0.86**(0.76–0.99)	0.90**(0.82–0.98)
Central	1.10(0.97–1.24)	1.01(0.90–1.13)	1.17***(1.09–1.26)
East	0.64***(0.55–0.74)	0.44***(0.39–0.50)	0.62***(0.57–0.67)
North East	1.17*(0.99–1.39)	0.91(0.78–1.05)	0.80***(0.72–0.88)
West	1.37***(1.16–1.62)	1.19**(1.02–1.38)	1.56***(1.40–1.73)

**Note**.

*p < 0.05

**p < 0.01

***p < 0.001.

Birth order of the children did not show consistently significant odds of dropout from different doses. Female children were 5% (AOR: 0.95, 95% CI: 0.89–1.00) less likely to get dropped out of the second dose and 3% (AOR: 0.97, 95% CI: 0.93–1.00) less likely to get dropped out of the third dose than the male children. Although mother’s educational attainment did not show consistently significant odds to dropout, still children of no educated mothers were 1.32 times more likely to get dropped out of the first dose, 1.45 times more likely to get dropped out of the second dose and 1.19 times more likely to get dropped out of the third dose than those children of higher educated mother. Wealth quintile showed very consistent and statistically significant odds associated with each of the dose specific dropout and the odds showed a gradual decreasing value from the poorer quintile to richer wealth quintile. Like, the dose specific missing, in case of dropout also, children from the poorest wealth quintile carried the highest odds of dropout for each of the doses. Children from the Eastern part of India showed the lowest odds of dropout for each of the doses whereas; children from the western part of India carried the burden of highest odds to dropout of different doses than the children from Southern India.

### Contribution of background characteristics in missing different hepatitis-B doses

**Table 5** presents Population Attributable Risk (PAR) estimates assessing the contribution of selected socio-economic and demographic factors in missing different hepatitis-B doses. All the background characteristics except sex of the child and place of residence demonstrated a statistically significant contribution towards missed birth dose. The PAR value was highest for institutional deliveries (PAR = -15.1%), which acted as a protective factor against missing birth dose. The children who hail from the Central region and missed their birth dose, 12.6% of missed birth dose could be attributed to the Central region. Similarly, children who hail from the Eastern region and missed their birth dose, 9.2% of missed birth dose could be attributed to the Eastern region. Similarly, children who hail from the Western region and missed their birth dose, 6.6% of missed birth dose could be attributed to the Western region. In addition, 6.2% and 4.8% reduction in missed birth dose can be achieved if individuals belonging to the poorest and poorer wealth quintile are eliminated, respectively.

**Table 5:** Population Attributable Risk (PAR) estimates for contribution of background characteristics in missing different doses of hepatitis-B among children aged 12–59 months, National Family Health Survey (NFHS-4), India, 2015–16.

Background characteristics	Birth dose	First dose	Second Dose	Third Dose
**Child’s age (in months)**				
12–23				
24–35	0.78***(0.37,1.18)		0.35*(-0.03, 0.73)	1.68***(1.23, 2.12)
36–47	2.13***(1.72, 2.53)	1.45***(1.11,1.80)	1.69***(1.30, 2.08)	4.40***(3.95, 4.84)
48–59	3.04***(2.63, 3.45)	2.93***(2.58, 3.28)	3.30***(2.91, 3.69)	7.15***(6.71, 7.58)
**Birth order**				
1				
2–3			0.34**(0.00, 0.68)	0.99***(0.61, 1.37)
4–5	0.36***(0.13, 0.59)	0.52***(0.30, 0.74)	0.68***(0.45, 0.92)	0.92***(0.68, 1.16)
6+	0.64***(0.50, 0.77)	0.77***(0.63, 0.91)	0.77***(0.63, 0.91)	0.63***(0.49, 0.77)
**Sex of the child**				
Male				
Female				
**Mother’s education**				
Higher Education				
No education	4.26***(2.95, 5.57)	6.89***(5.68, 8.10)	7.64***(6.38, 8.90)	6.60***(5.33, 7.86)
Primary or less	1.26**(0.26, 2.26)	1.40***(0.53, 2.28)	2.21***(1.27, 3.15)	1.44***(0.40, 2.47)
Secondary or less			0.95*(-0.07, 1.97)	
**Social group**				
Non-Scheduled Castes/Tribes				
Scheduled Castes		-0.34***(-0.53, -0.16)	-0.33***(-0.54, 0.13)	-0.45***(-0.69,-0.22)
Scheduled Tribes	-0.54***(-0.73, 0.35)	-0.24***(-0.41, -0.08)	-0.21**(-0.39, 0.02)	-0.16*(-0.35, 0.02)
**Religion**				
Hindu				
Muslim	0.98***(0.76, 1.21)	0.75***(0.56, 0.94)	0.82***(0.61,1.03)	0.72***(0.49, 0.95)
Christian		0.05*(0.00, 0.10)	0.05*(-0.01, 0.12)	
Others	-0.30***(-0.38, -0.22)	-0.23***(-0.29, -0.16)	-0.31***(-0.38, -0.25)	-0.51***(-0.59, -0.43)
**Wealth quintiles** ^**£**^				
Richest				
Poorest	6.15***(4.95, 7.36)	7.62***(6.66, 8.59)	8.26***(7.25, 9.27)	8.88***(7.78, 9.98)
Poorer	4.75***(3.73, 5.78)	4.33***(3.52, 5.13)	4.54***(3.69, 5.39)	4.92***(3.94, 5.89)
Middle	3.21***(2.36, 4.07)	2.51***(1.83, 3.18)	2.71***(1.95, 3.46)	3.12***(2.24, 3.99)
Richer	1.98***(1.20, 2.76)	1.51***(0.89, 2.12)	2.07***(1.34, 2.80)	2.25***(1.38, 3.12)
**Place of Residence**				
Urban				
Rural			-1.01**(-1.84, -0.19)	-1.48***(-2.38, -0.59)
**Place of delivery**				
Home				
Institutional	-15.14***(-15.86, -14.43)	-7.41***(-8.01, -6.81)	-7.86***(-8.51, -7.21)	-6.73***(-7.44, -6.03)
**Region**				
South				
North	3.47***(2.84, 4.10)	2.22***(1.70, 2.74)	1.61***(1.01, 2.20)	0.57(-0.13, 1.27)
Central	12.56***(11.71, 13.41)	3.84***(3.08, 4.59)	3.09***(2.26, 3.91)	3.10***(2.28, 3.91)
East	9.18***(8.27, 10.10)	-1.53***(-2.32, -0.75)	-4.20***(-5.06, -3.34)	-7.07***(-7.94, -6.20)
North East	5.72***(5.42, 6.01)	1.93***(1.67, 2.20)	1.53***(1.24, 1.82)	0.61***(0.33, 0.89)
West	6.63***(5.83, 7.43)	4.87***(4.20, 5.55)	4.76***(4.04, 5.49)	5.95***(5.12, 6.78)

**Note**.

*p< 0.05

**p < 0.01

***p < 0.001. The table shows the statistically significant PAR estimates only.

Similar to birth dose, all the background variables except sex of the child presented a statistically significant contribution on missing the first dose among the children. The findings suggest that 7.6% and 4.3% reduction in missed first dose can be achieved if individuals belonging to the poorest and poorer wealth quintile are eliminated, respectively. The PAR value was highest for institutional deliveries (PAR = -7.4%), which acted as a protective against missing first dose. The PAR value for the education suggests a 6.9% reduction in missed first dose can be achieved if illiteracy (no education) were eliminated, respectively.

For missed second dose, 8.3% and 4.5% reduction can be achieved if individuals belonging to the poorest and poorer wealth quintile are eliminated, respectively. The PAR value was highest for institutional deliveries (PAR = -7.9%), which acted as a protective against missing second dose. The PAR value for the education suggests a 7.6% reduction in missed second dose can be achieved if illiteracy (no education) were eliminated, respectively. In case of the missed third dose, 8.8% and 4.9% reduction can be achieved if individuals belonging to the poorest and poorer wealth quintile are eliminated, respectively. The PAR value for the child’s age suggests a 7.2% reduction in missed third dose can be achieved if children aged 48–59 months are eliminated, respectively. In addition, Eastern region acted as a protective sub-group (PAR = -7.1%) for missing third dose of hepatitis- B vaccination.

### Contribution of background characteristics in dropout of different hepatitis-B doses

**Table 6** presents PAR estimates for assessing the contribution of socio-economic and demographic variables in hepatitis-B dose dropout. Background characteristics child’s age (in months), birth order, mother’s education, wealth quintile, and region presented a statistically significant contribution for dropout between birth dose and first dose. The findings suggest that 1.5% and 1.2% reduction in first dose dropout can be achieved if individuals belonging to the poorest and poorer wealth quintile are eliminated, respectively. In case of dropout between first and second dose, variables child’s age, birth order, sex of child, mother’s education, wealth education, place of residence, and region presented a statistically significant contribution. The findings suggest that Eastern (PAR = -2.9%), Northern (PAR = -0.4%), and North-eastern (PAR = -0.1%) region acted as protective against second dose dropout. Furthermore, a reduction of 1.6% in second dose dropout can be achieved if individuals belonging to the poorest wealth quintile are eliminated. For dropout between second and third dose, all background variables presented a statistically significant contribution. The findings suggest that Eastern (PAR = -4.7%), Northern (PAR = -0.7%), and North-eastern (PAR = -0.5%) region acted as protective sub-group against third dose dropout.

**Table 6:** Population Attributable Risk (PAR) estimates for contribution of background characteristics in hepatitis-B dose dropout among children aged 12–59 months, National Family Health Survey (NFHS-4), India, 2015–16.

Background characteristics	Dropout (0–1)	Dropout (1–2)	Dropout (2–3)
**Child’s age (in months)**			
12–23			
24–35	-0.54***(-0.78, -0.29)	0.31**(0.04, 0.59)	1.90***(1.46, 2.33)
36–47		0.42***(0.15, 0.69)	4.07***(3.63, 4.52)
48–59		0.63***(0.37, 0.89)	6.09***(5.64, 6.53)
**Birth order**			
1			
2_3		0.25**(0.01, 0.49)	0.89***(0.52, 1.27)
4–5		0.23***(0.06, 0.39)	0.51***(0.28, 0.74)
6+	0.07*(0.00, 0.14)	0.07*(0.00, 0.15)	
**Sex of the child**			
Male			
Female		-0.18*(-0.36, 0.00)	-0.28*(-0.59, 0.03)
**Mother’s education**			
Higher Education			
No education	1.07***(0.44,1.69)	1.79***(1.04, 2.54)	2.30***(1.11, 3.49)
Primary or less		1.18***(0.59, 1.77)	
Secondary or less			-1.52***(-2.65, -0.38)
**Social group**			
Non-Scheduled Castes/Tribes			
Scheduled Castes			-0.27**(-0.50, -0.05)
Scheduled Tribes			
**Religion**			
Hindu			
Muslim		0.15**(0.03, 0.28)	
Christian			
Others	-0.09***(-0.14, -0.04)	-0.13***(-0.16, -0.09)	-0.38***(-0.46, -0.29)
**Wealth quintiles** ^**£**^			
Richest			
Poorest		1.62***(1.10, 2.13)	
Poorer		0.70***(0.22, 1.18)	1.94***(1.11,2.77)
Middle		0.46*(0.00, 0.93)	1.36***(0.58, 2.15)
Richer		0.82***(0.25, 1.39)	0.86**(0.09, 1.63)
**Place of Residence**			
Urban			
Rural		-0.43*(-0.88, 0.01)	-0.87**(-1.69, -0.05)
**Place of delivery**			
Home			
Institutional		-1.16***(-1.57, -0.75)	-0.72*(-1.45, 0.02)
**Region**			
South			
North	0.42***(0.16, 0.69)	-0.39**(-0.74, -0.03)	-0.72**(-1.33, -0.10)
Central			1.66***(0.91, 2.41)
East	-1.10***(-1.48,-0.72)	-2.91***(-3.46, -2.36)	-4.71***(-5.52, -3.90)
North East	0.08*(-0.01, 0.16)		-0.52***(-0.73, -0.30)
West	0.62***(0.28, 0.96)	0.48**(0.06, 0.90)	3.17***(2.41, 3.94)

**Note**.

*p < 0.05

**p < 0.01

***p < 0.001. The table shows the statistically significant PAR estimates only.

### Bayesian spatial estimation

A series of district level interactive maps of SIR estimates for all the outcome variables were shown in **S1 Fig.** These maps demonstrated a gradual spatial progression from low to high risk of dose specific missing and drop out of hepatitis-B vaccine across districts of India. For almost all the outcome variables, districts with higher SIR value were clustered mostly in the North-Eastern, Central and Northern regions of India. States like West Bengal (WB) and Bihar from Eastern part, Maharashtra from Western part and Telangana (TG) & Andhra Pradesh (AP) from Sothern part included districts with lesser number of missing and dropout cases than expected.

**S8 Table** presents the fitted model outputs, model diagnostics and covariate specific estimates of posterior median and 95% credible interval for the missing different doses of hepatitis- B vaccination. **S9 Table** illustrates the fitted model outputs, model diagnostics and covariate specific estimates of posterior median and 95% credible interval for dropout of hepatitis-B. The CARLeroux Bayesian model with the lowest DIC value provided the final estimates for each of the outcome variables under study. We generated MCMC samples from three independent Markov chains to check the model convergence and to draw final inferences. The sample specific trace plots for each of the parameters and the Gelman-Rubin test statistic (a value less than 1.1 depicts a good mixing of the chain) suggested model convergence for all the outcome variables.

**Fig 1:**
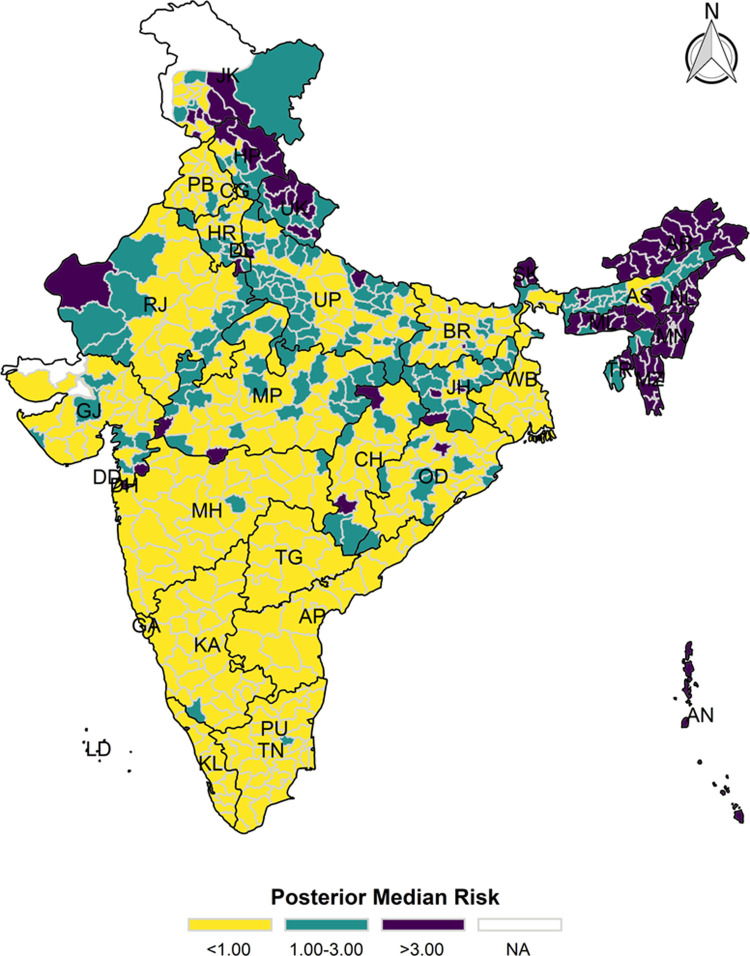
Spatial distribution of Posterior Median Risk (PMR) of missing birth across 640 districts of India (https://github.com/datameet/maps/), NFHS-4, 2015–16.

**Fig 2:**
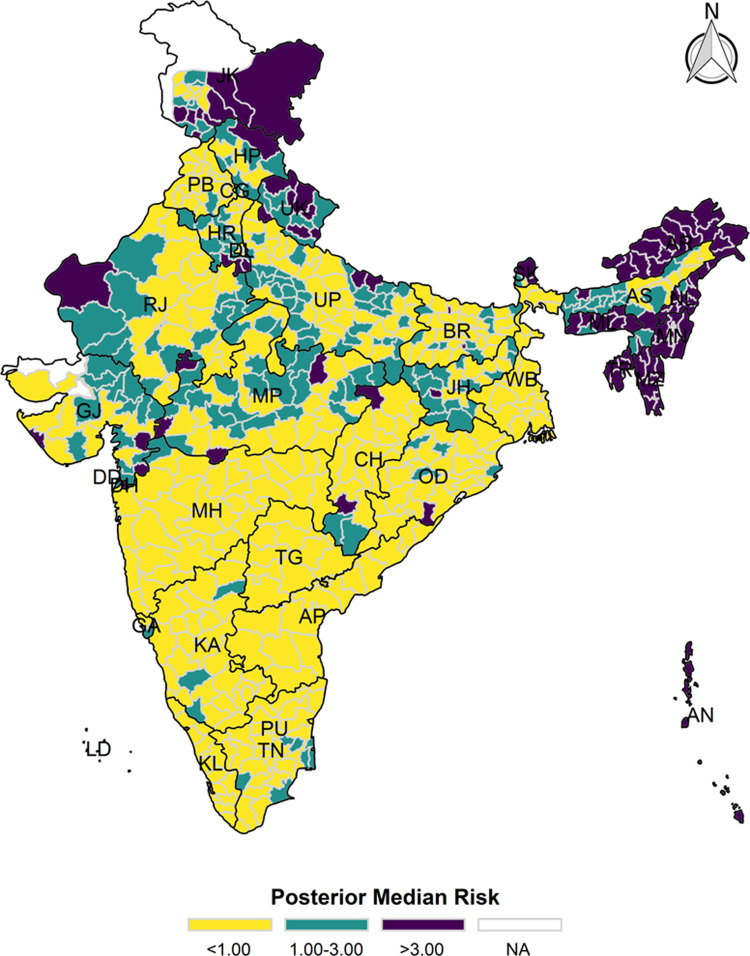
Spatial distribution of Posterior Median Risk (PMR) of missing first dose across 640 districts of India (https://github.com/datameet/maps/), NFHS-4, 2015–16.

**Fig 3:**
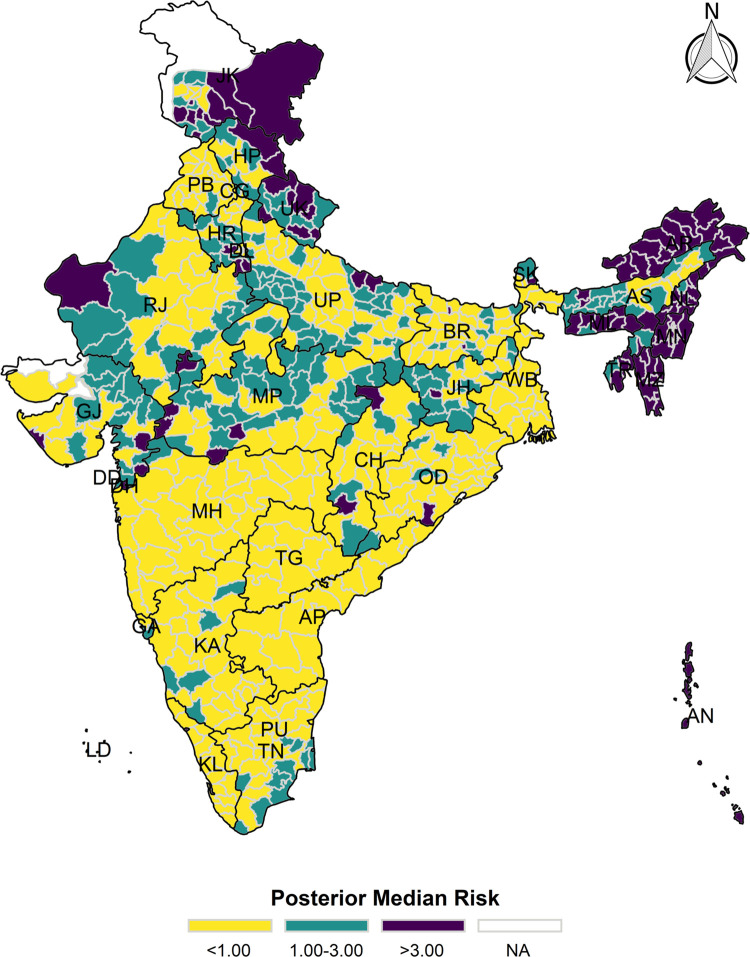
Spatial distribution of Posterior Median Risk (PMR) of missing second dose across 640 districts of India (https://github.com/datameet/maps/), NFHS-4, 2015–16.

**Fig 4:**
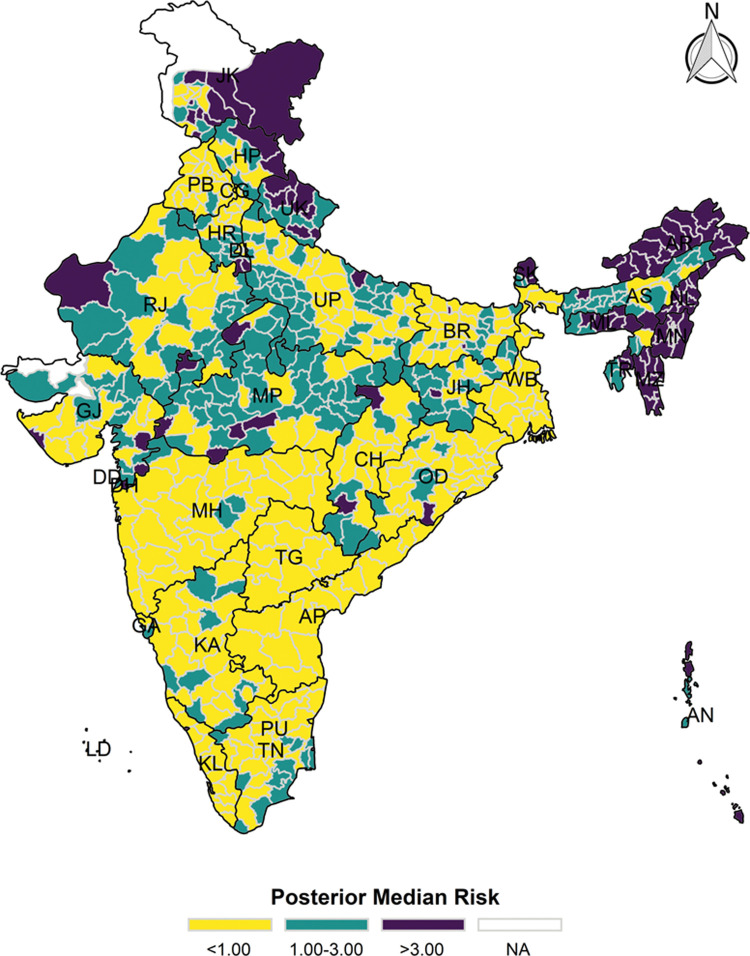
Spatial distribution of Posterior Median Risk (PMR) of missing third dose across 640 districts of India (https://github.com/datameet/maps/), NFHS-4, 2015–16.

**Fig 5:**
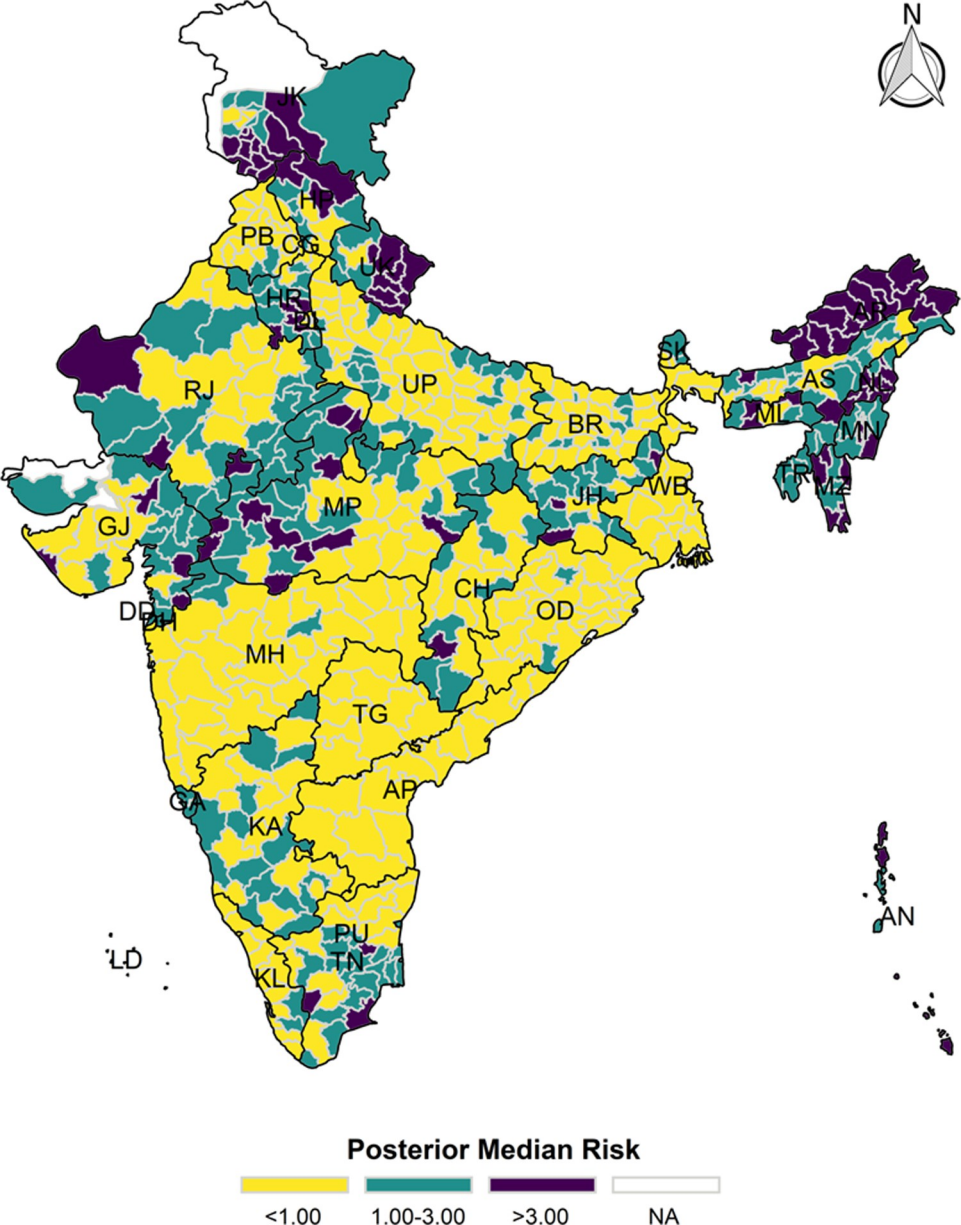
Spatial distribution of Posterior Median Risk (PMR) of first dose dropout across 640 districts of India (https://github.com/datameet/maps/), NFHS-4, 2015–16.

**Fig 6:**
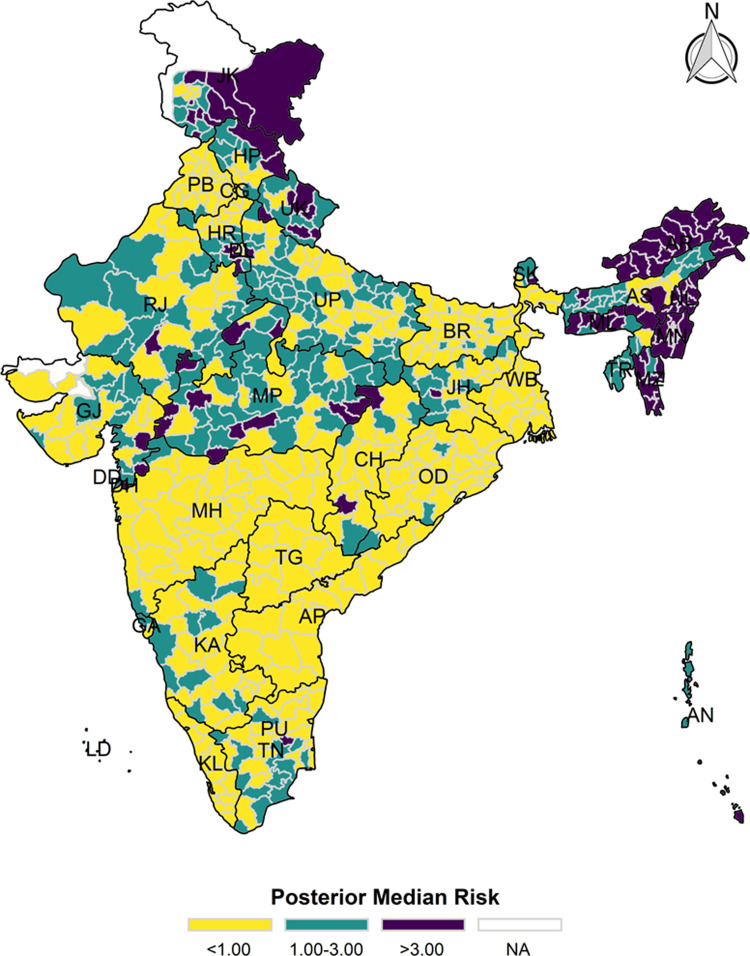
Spatial distribution of Posterior Median Risk (PMR) of second dose dropout across 640 districts of India (https://github.com/datameet/maps/), NFHS-4, 2015–16.

**Fig 7:**
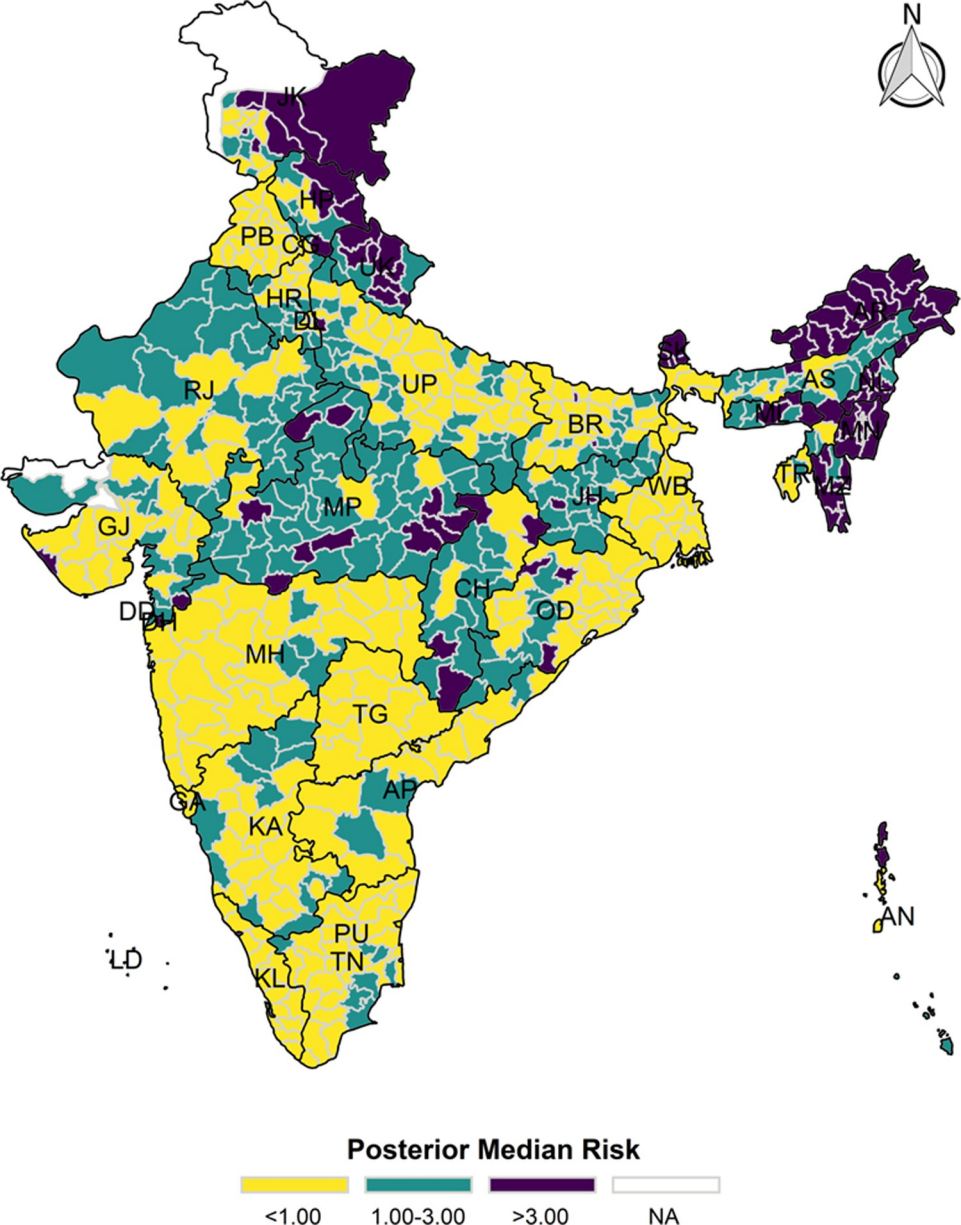
Spatial distribution of Posterior Median Risk (PMR) of third dose dropout across 640 districts of India (https://github.com/datameet/maps/), NFHS-4, 2015–16.

**Fig 8:**
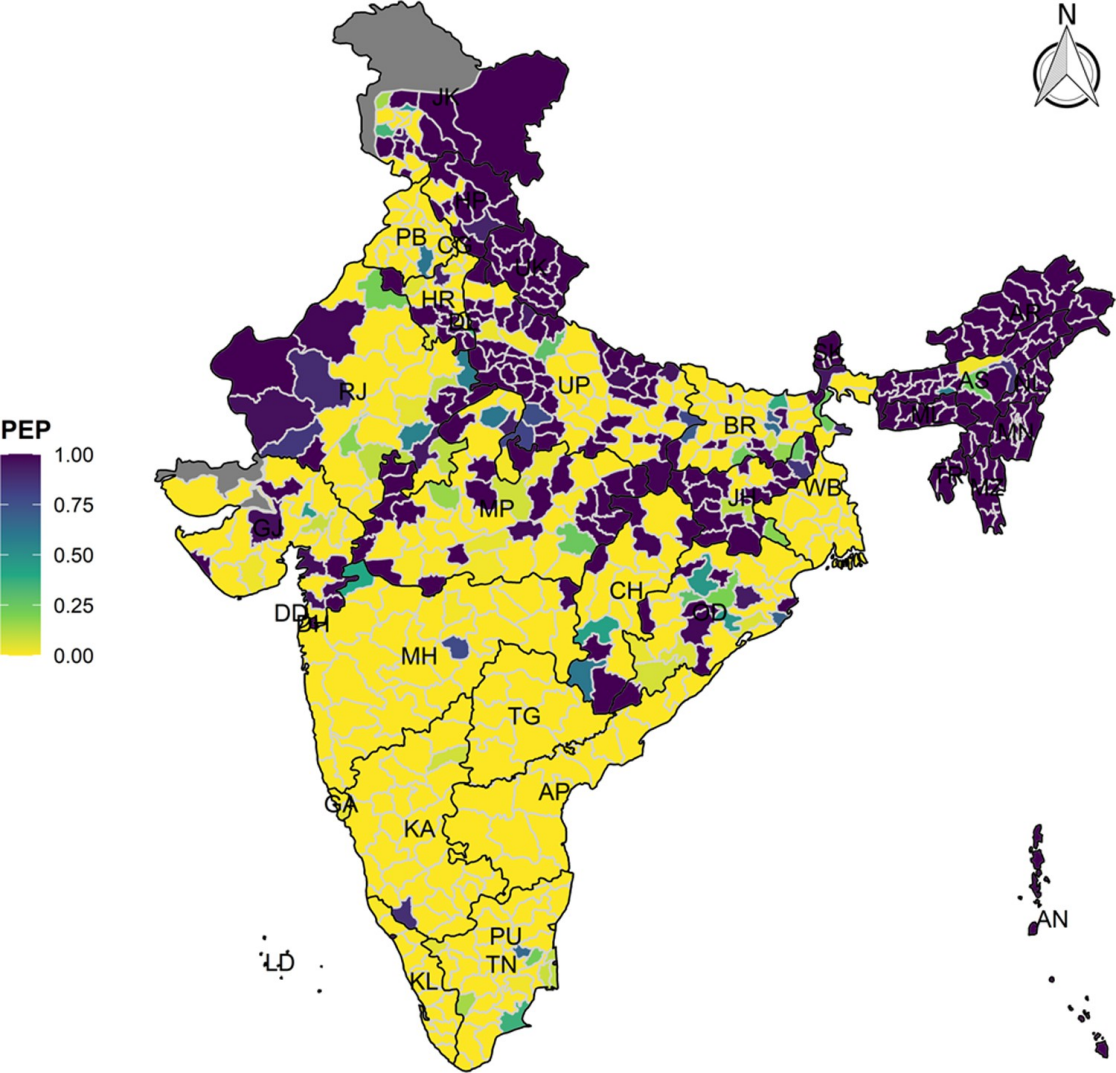
Spatial distribution of Posterior Exceedence Probability (PEP) of missing birth dose across 640 districts of India (https://github.com/datameet/maps/), NFHS-4, 2015–16.

**Fig 9:**
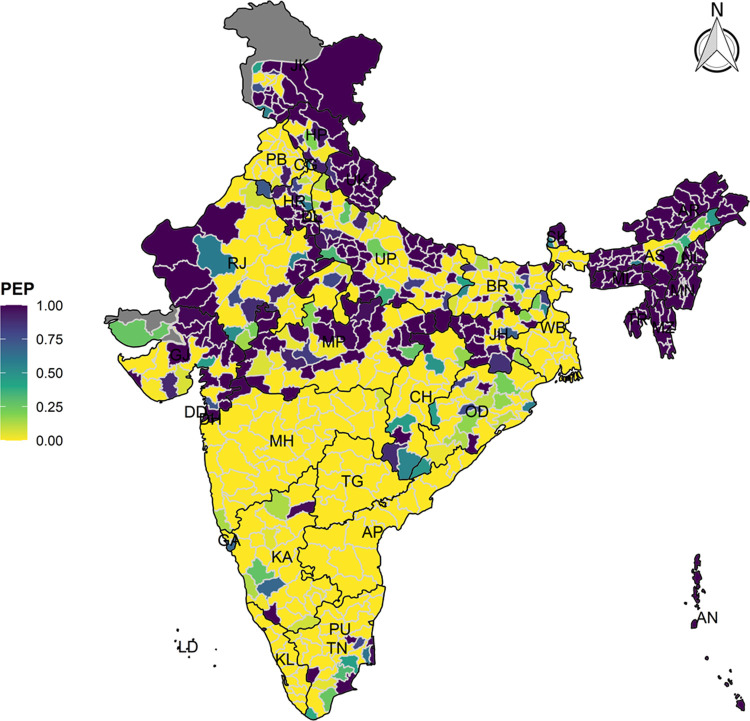
Spatial distribution of Posterior Exceedence Probability (PEP) of Missing First Dose across 640 districts of India (https://github.com/datameet/maps/), NFHS-4, 2015–16.

**Fig 10:**
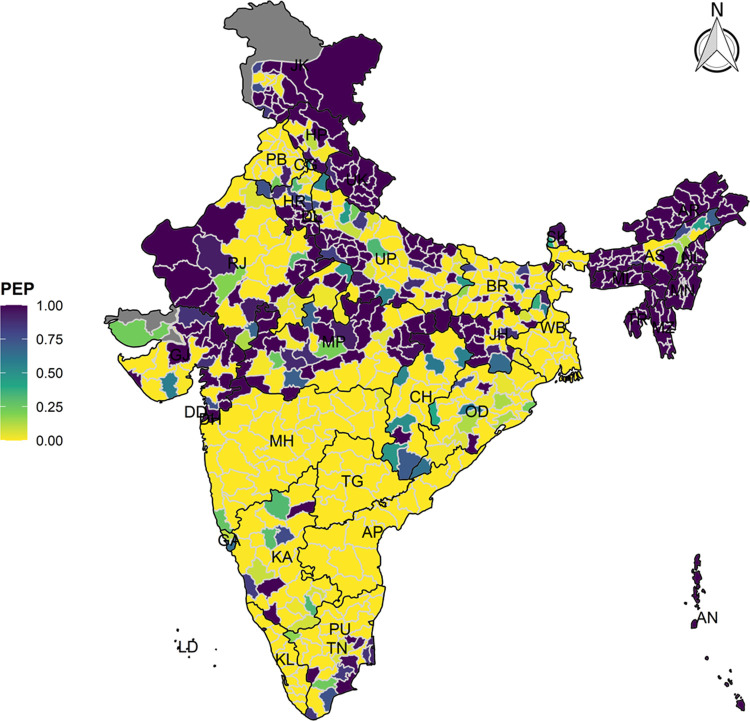
Spatial distribution of Posterior Exceedence Probability (PEP) missing second dose across 640 districts of India (https://github.com/datameet/maps/), NFHS-4, 2015–16.

**Fig 11:**
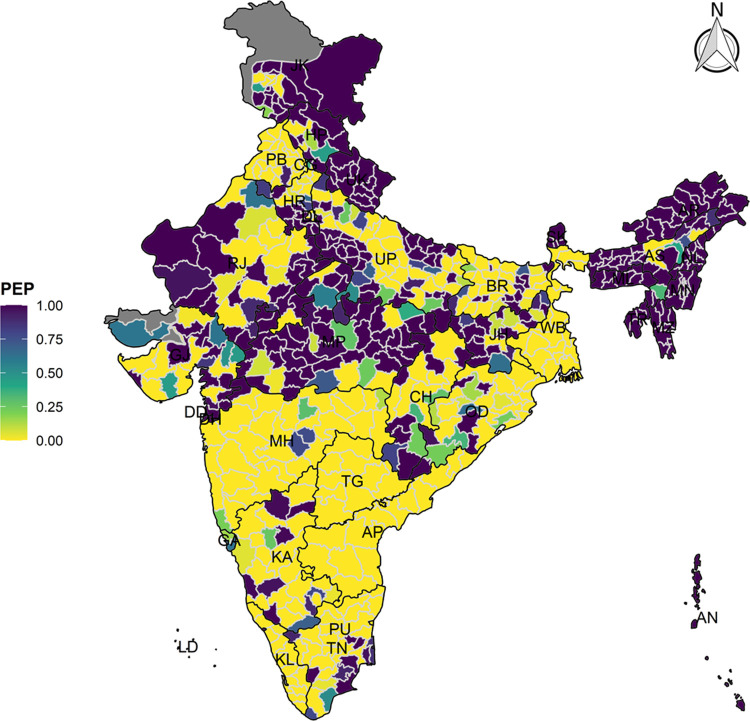
Spatial distribution of Posterior Exceedence Probability (PEP) of missing third dose across 640 districts of India (https://github.com/datameet/maps/), NFHS-4, 2015–16.

**Fig 12:**
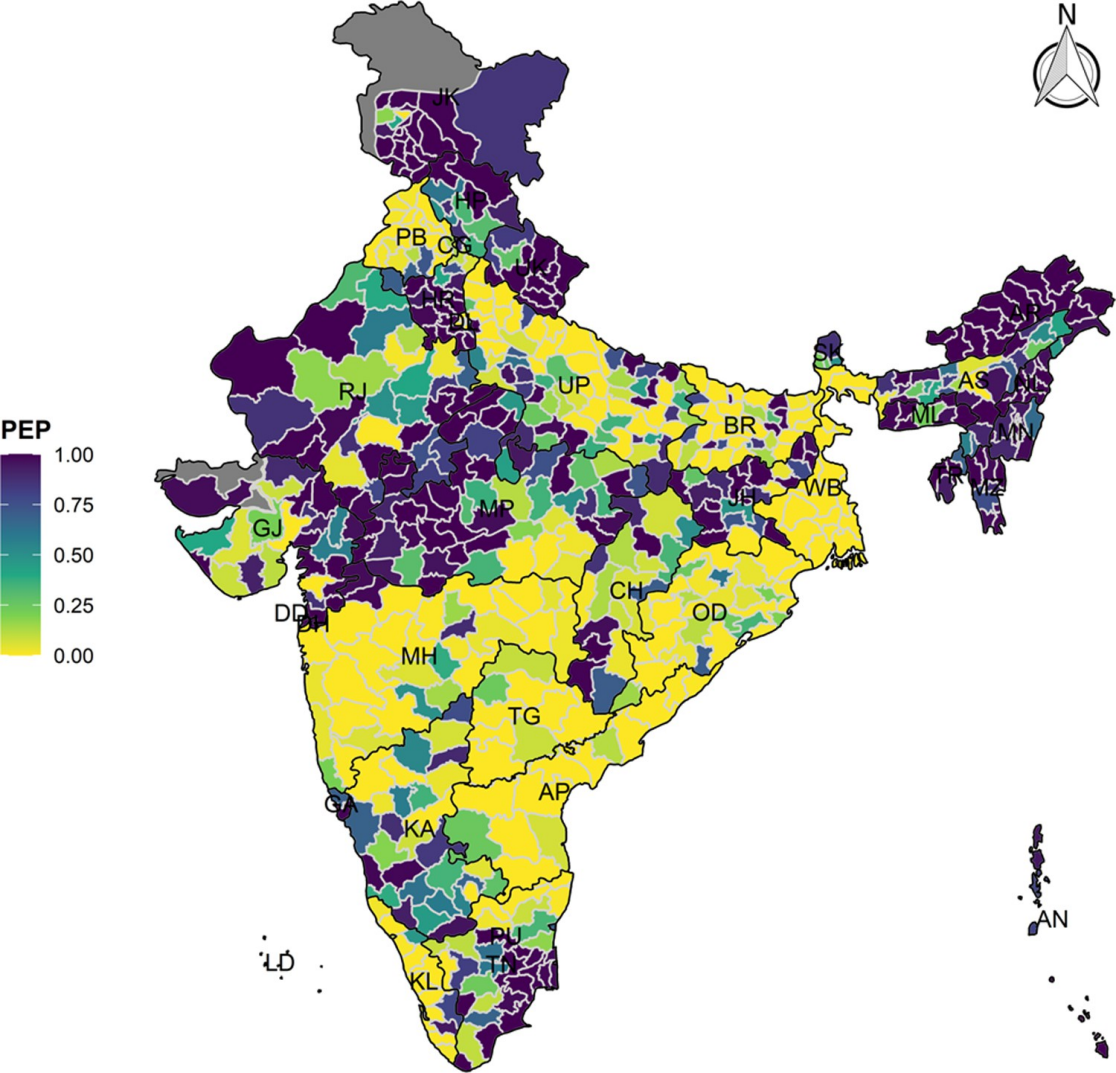
Spatial distribution of Posterior Exceedence Probability (PEP) of first dose dropout across 640 districts of India (https://github.com/datameet/maps/), NFHS-4, 2015–16.

**Fig 13:**
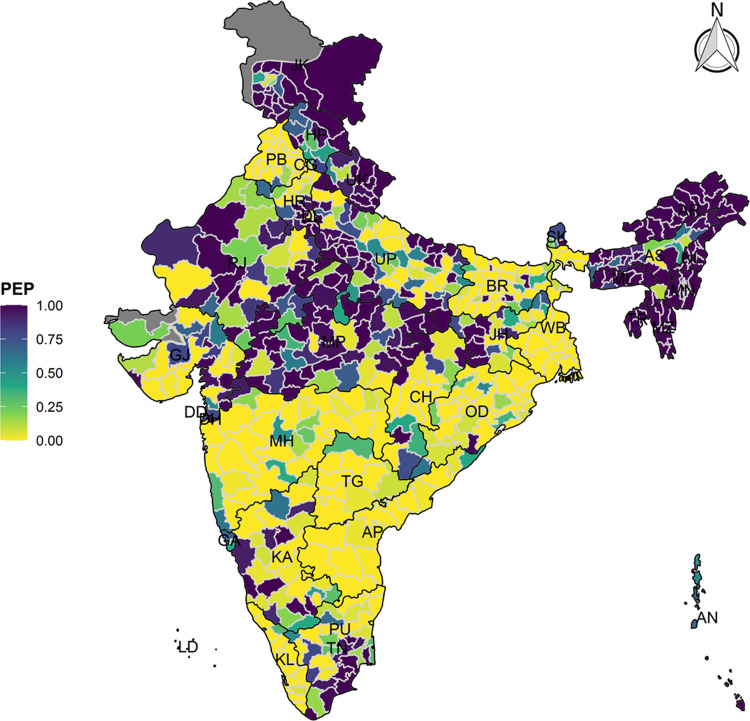
Spatial distribution of Posterior Exceedence Probability (PEP) of second dose dropout across 640 districts of India (https://github.com/datameet/maps/), NFHS-4, 2015–16.

**Fig 14:**
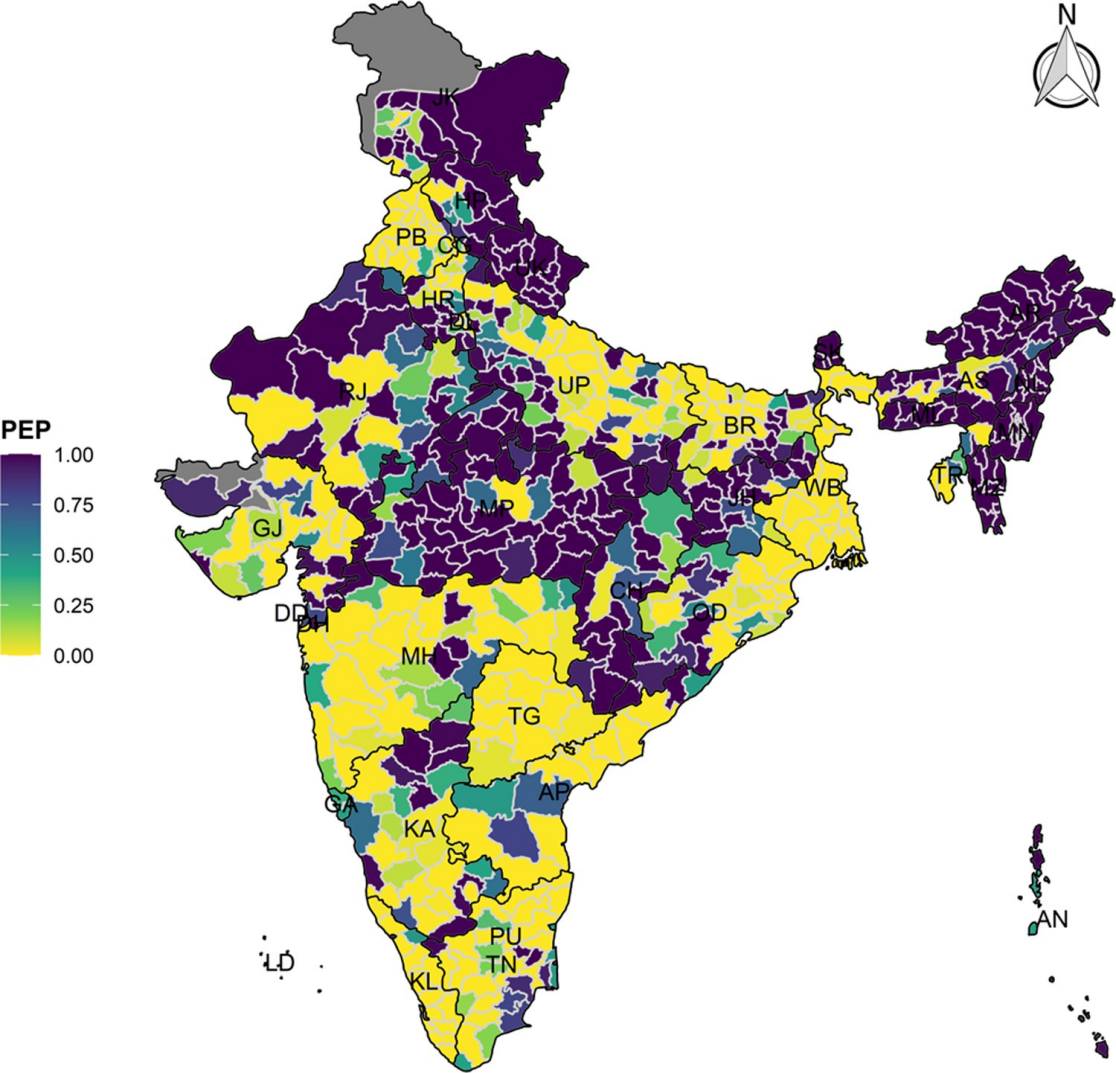
Spatial distribution of Posterior Exceedence Probability (PEP) of third dose dropout across 640 districts of India (https://github.com/datameet/maps/), NFHS-4, 2015–16.

The model-based district level estimates of PMR were mapped **Figs 1–7**. In addition, the model-based district level estimates of PEP were mapped **Figs 8–14**. The maps demonstrated that around 282 districts across India carried higher risk (risk>1) to miss the birth dose, 284 districts to miss the first dose, 303 districts to miss the second dose, 332 districts for third dose, 317 districts of first dose dropout, 315 districts of second dose dropout and 339 districts of third dose dropout. A comparatively higher PMR (for all the outcome variables) was observed in districts mostly from the North-Eastern States (e.g., Assam, Meghalaya, Manipur, Nagaland, Mizoram, Sikkim, and Arunachal Pradesh), Northern states (e.g., Uttarakhand, Jammu and Kashmir, Haryana, Himachal Pradesh) other states/UTs including Uttar Pradesh (Central region), Rajasthan (Western region), Jharkhand (Eastern region), Madhya Pradesh (Central region), Tamil Nadu (Southern region), Karnataka (Southern region).

State wise list of districts with higher PMR for all the outcome variables were shown in **S10 Table** (missing dose) and **S11 Table** (drop out). According to district level PEP estimates, we observed a significant number of districts with more than 75% exceedance probability. Of the total 640 districts, 271 districts carried more than 75% PEP to miss the birth dose. Similarly, 267 districts in terms of missing the first dose, 283 districts to miss the second dose, 313 districts to miss the third dose. In terms of dropout, 272 districts for the first dose, 271 districts for the second dose and 304 districts for the third dose carried more than 75% exceedance probability.

## Discussion

The findings from the present study suggest that missing and dropout of different doses of the hepatitis-B vaccine is one of the challenging public health concerns in India. The range of this issue varies largely at the sub-national level (across districts) and across different sub-population of the country. In 2015–16, the national average of missing the birth dose was 38% (almost two-fifth of the children) among the children aged 12–59 months. Markedly, this prevalence was highest (44%) for the third dose, followed by the second and the first dose. The dropout rate is also significant among the children and those who missed the birth dose; 58% did not complete the full immunization schedule. Notably, around 6% of the children who received the birth dose were dropped out of the first dose. The dropout rate was relatively high for the third dose. The global prevalence of hepatitis-B infection among children under age five is estimated to be 1.3% [27]. India contributed to around 17 million chronic hepatitis B carriers with a prevalence of 1.46% [28, 29]. In terms of Hepatitis-B surface antigen (HBsAg), India belongs to the “intermediate to high endemicity” group of countries, and childhood immunization against hepatitis-B remains the most effective way to prevent the infection among the children, including the birth dose within 24 hours of birth, which is preventive against the perinatal transmission among the new born [6].

This study demonstrates the level and patterns of missing and dropout of different doses of hepatitis-B in India. Missing the birth dose is substantially high among Indian children, and the prevalence varies largely by socio-economic and demographic characteristics of the children. In this regard, it is observed that the prevalence is lop-sided high among children of higher birth order (4–5, 6+). Similarly, more than two-thirds of the children delivered in a home (non-institutional setting), did not receive the birth dose. The prevalence of missing the birth dose was also very high among children from North-east region. Like, the event of missing different doses, dropout is also very prominent among Indian children, and there exists a predominant socio-economic and demographic pattern of dropout rates.

This study examined the likelihood of missing and dropout of different doses of hepatitis-B among the comparison groups. It provided a detailed understanding of the determinants of missing and dropout of different doses of hepatitis-B. Age-wise, older children carry a statistically significant higher likelihood to miss the birth dose, and other consecutive doses of hepatitis-B compared to the younger children. This suggested that children born earlier were less likely to receive birth dose and other doses of hepatitis-B. A child’s birth order is one of the variables significantly associated with the missing birth and other doses. Children of higher birth order are always more likely to miss different doses than children of lower birth order.

Previous studies found the linkages of birth order and utilization of immunization services by the parents for their children where it is established that with more experience to child-rearing, parents develop the confidence to dismiss the importance of health care refusing to go for immunization for their later-born child. It is also suggested that parents refuse to go for immunization for their later-born child subject to their previous experience of adverse effects of immunization on their earlier-born child [30, 31]. This study also exhibits a higher likelihood of missing different doses of hepatitis-B among the higher birth-ordered children. The sex of the child does not show any statistically significant association with missing different doses. Children born to higher educated mothers are less likely to miss the birth dose and the other doses of hepatitis-B. In this regard, previous studies found that educated mothers know the importance of immunization and vaccinating their children against vaccine-preventable diseases [32–34].

Among different social groups, children from the Scheduled Tribes (ST) class show a comparatively lower likelihood of missing different doses of hepatitis-B. Among different religious groups, Muslim children carry a higher likelihood to miss the birth dose as well as the other doses of hepatitis-B. Household’s economic status demonstrated a robust statistically significant association with the vaccination status of the children. Children from the poorest and poorer wealth quintile carry a substantially higher likelihood of missing the birth dose and the other doses of hepatitis-B. It is already studied that there is a large pro-poor disparity in immunization coverage and the rich class is always at the advantage of better immunization coverage [35]. From this study also, it is evident that children from economically poor households are vulnerable and carry a higher risk of missing the doses of hepatitis-B.

Adjusted population-attributable risks (PARs), derived from the multivariable logistic regression model for each of the independent risk factors, indicated the proportions of the children associated with events of missing and dropout of different doses of hepatitis-B. These PAR proportions provide the estimates of the magnitude of missing and dropout in the study population. Subject to different socio-economic and demographic risk factors, the PAR proportions associated with the missing hepatitis-B doses are observed higher than the dropout of hepatitis-B doses in the study population. And the magnitude was relatively high (birth and other consecutive doses) among children of the uneducated mother, children from the poorest economic background (wealth quintile), those who were born in-home (non-institutional setting), and children who belong to the Central and Western parts of India. The bivariate, as well as the multivariable estimation confirmed a strong socio-economic and demographic pattern in missing as well as in dropout of different doses of hepatitis-B. Though the missing prevalence and dropout rates are not directly comparable to each other when considered the previous dose, being dropped out of the next dose is comparatively less common in the study population.

The district-level spatial estimation helps to identify those districts with a higher risk of missing different doses of hepatitis-B and those districts that carry substantial risk in terms of drop out of different doses. Using the estimated posterior median risk, this study further investigates the exhaustive list of districts with higher PMR (PMR>1) in every state/UTs of India (**S9 Table**). It thereby informs to prioritize the vaccination drive and targets those districts to increase the coverage of three doses of hepatitis-b vaccine. The majority of the districts from the states like Assam, Himachal Pradesh, Jharkhand, Manipur, Meghalaya, Mizoram, Nagaland, Sikkim, Tripura and Uttarakhand demonstrate a predominantly higher PMR in terms of missing the birth dose, which needs careful attention to prevent the perinatal transmission among the new-born because it is estimated that infection during childhood leads to death in adulthood in 15–25% of the cases [9]. At the same time, in India, the maternal screening of hepatitis B surface antigen (HBsAg) is not well-implemented. Thus, it is crucial to ensure the birth dose and the other consecutive doses for optimum protection against the hepatitis-B infection.

Like other health interventions, paediatric immunization against all the vaccine-preventable diseases, including hepatitis-B is one of the major SDG targets as well as the target of the WHO-Global Health Sector Strategy (GHSS) to increase the vaccine coverage globally and reduce the hepatitis-B infection-associated deaths by 10% [9], and identifying the public health threat in terms of viral hepatitis infection, the GHSS, 2016–2021 by the World Health Assembly (WHA) aim to reduce new infections by 90% and mortality by 65% [9].

India has a mixed health care system consisting of public and private health care providers. Concentrated primarily in the urban parts, private health care providers mainly provide secondary and tertiary health care facilities while the public health care infrastructure with a three-tier system-sub center (SC), primary health center (PHC) & community health center (CHC) provides health care facilities across the country [36]. As PHCs and SCs serve as the primary level Human Resources for Health Unit (HRH) in the country, they are equipped and assigned to immunize all the children under age five against vaccine-preventable diseases (VPDs) [37, 38].

Under the Universal Immunization Programme (UIP) in India, a routine childhood immunization programme rolls out and is primarily provided at SCs through static and outreach sites. In India, during the COVID-19 pandemic, preventive health care services like child immunization faced an unprecedented disruption holding on this primary health care service and putting the women and children at the risk of VPDs like measles, rotavirus and tetanus (39). COVID-19 pandemic preparedness caught all the attention to meet the public health emergency affecting various on-site and community immunization services across India. In April 2020, the Health Management and Information System (HMIS) data confirmed a decrease in the total number of routine immunization sessions and the number of fully immunized children [39].

To guide the paediatricians, Advisory Committee on Vaccines and Immunization Practices (ACVIP) in India took an initiative to address the issues associated with routine immunization and COVID-19. ACVIP recommended no change in the immunization schedule and depending upon the WHO guidelines on area categorization subject to active COVID-19 cases, the immunization services were provided in alignment with the area categorization, and the services were classified into two heads- immunization in containment and buffer zones and immunization in areas beyond buffer zones and green zones [39]. While recommending how to conduct the immunization services, the ACVIP recommended the birth dose at all the health facilities and guided the immunization services to follow certain principles and thereby instructed to administer the birth dose of hepatitis-B within 24 hours of birth along with OPV and BCG vaccine or otherwise at the first contact with the healthcare facility [39].

Multiple risk factors influence the likelihood of being completely vaccinated against hepatitis-B among Indian children [6]. In this direction, this study is a benchmark to provide a detailed understanding of the relative contribution of multiple socio-economic and demographic risk factors of missing and dropout of different doses of hepatitis-B within a cross-sectional framework. Additionally, the Bayesian spatial analyses helped identify the set of districts with an elevated risk of missing and dropout of different hepatitis-B doses utilizing the data from the recent round of the NFHS survey.

However, there was no data available on child immunization against hepatitis-B until the fourth round of the NFHS survey conducted during 2015–16, a survey providing crucial estimates on the coverage of different doses of the full immunization schedule to monitor the immunization coverage among under-five children over time. NFHS, during its fourth round, collected the necessary information on hepatitis B vaccination for the first time. And the survey remains the only source to get sub-national level (district) estimates of hepatitis-B in recent times.

There are three different modes in India where immunization services are provided; birth dose vaccination, health facility-based sessions and outreach sessions. The birth doses of different vaccines, including hepatitis-B, are traditionally provided at the delivery [40] points, but many births in India occur in the home, a non-intuitional setting, in the absence of a skilled birth attendant/doctor without any health care facility. Like other countries, administering a birth dose of hepatitis B remains a challenge when many births happen in a non-institutional setting [40]. Considering the non-institutional births in India, three doses are administered at 6, 10, and 14 weeks irrespective of the birth dose, as per the national immunization schedule followed in the country [41]. In India, under the UIP, the different doses of full immunization, including hepatitis-B doses, are provided free of cost in public health facilities; still, 38% (**Table 1**) of the children did not receive the birth dose, and 45% [10] of the child did not complete the full immunization schedule of hepatitis-B.

It is studied that any interruptions to routine immunization services may cause an outbreak of vaccine-preventable diseases and the associated morbidity and mortality [42]. And since early 2020, the COVID-19 pandemic has affected the routine immunization services in India. Though India’s immunization programme is the largest globally in terms of beneficiaries, still almost two-fifths of the children did not complete all the basic vaccine doses in their first year of life in 2016 [10, 42, 43]. Taking care of the low vaccination coverage, the Ministry of Health and Family Welfare, Government of India launched the Mission Indradhanush (MI) programme in 2014 to increase vaccination coverage among the vulnerable, underserved, resistant and inaccessible populations. Notably, this immunization programme increased the full immunization coverage among children by 6.7% during April 2015 and July 2017 [44, 45]. Further, in October 2017, the Intensified Mission Indradhanush (IMI) strategized to improve the immunization coverage by 90% in those districts and in urban areas where the coverage was persistently low [45]. In 1991, the Extended Program on Immunization (EPI) recommended introducing the vaccine doses of Hepatitis-B in the routine program immunization. By 2009, about 175 WHO member countries could integrate hepatitis-B vaccination in their National Immunization Program [45, 46]. India launched the hepatitis-B vaccination in 2002, and finally, after 2003, the Indian National Policy on Immunization recommended vaccinating the children with three doses of hepatitis-B along with the other doses of routine immunization [47].

This study identifies the population sub-groups with the substantially higher attributable risk associated with missing birth and other consecutive doses. Place of delivery shows the highest PAR associated with the birth dose and for the rest of the doses. This suggests that children born in non-institutional settings are at the highest risk of missing birth and subsequent doses. Other than the place of delivery, mother’s education (no education), wealth status (poorest & poorer wealth quintile) also shows high attributable risk associated with missing the birth and other doses of hepatitis-B vaccine. The regional pattern of PAR estimates indicates that the attributable risk of missing the birth dose is highest among the children from the Central region, followed by East, West, and North-East. This study also identified the sub-groups with the higher attributable risk associated with the dropout of different doses. In this context, it is noteworthy to mention that the Intensified Mission Indradhanush (previously Mission Indradhanush), launched in October 2017, targeted those areas with higher rates of immunization dropouts in terms of diphtheria, tetanus, pertussis 3 (DPT3)/pentavalent 3 in the previous year and not certainly considered hepatitis-B vaccination dropout among Indian children. So, the estimation of population attributable risk of missing and dropout of different doses for the population sub-groups essentially contributes to the understanding related to the intensity of dose-specific poor coverage of hepatitis-B vaccination among Indian children under age five.

This study demonstrates the status of missing patterns and dropout patterns of different doses of hepatitis-B vaccination in India, i.e., across its diverse sub-populations and districts. Although India has shown an improvement in terms of vaccination coverage, large number of children do not receive the birth dose of hepatitis-B. An alarmingly significant portion of the children fails to complete the full immunization schedule of hepatitis B. And eventually, the risk of missing the birth dose and other consecutive doses is also very high in some districts and in some of the sub-populations. Although the vertical transmission is low, horizontal transmission largely contributes to the hepatitis-B infection in India [48]. It is essential to scale up the immunization coverage of hepatitis B and the other doses of full immunization protecting the children from VPDs, including hepatitis infection, for their lifetime. During 2011–20, the GVAP set a target for the childhood vaccination coverage to reach 90% across all vaccine doses, and the Immunization Agenda 2030 set a goal for equitable access for all the routine vaccines for every child and halving the number of zero-dose children missed by the current vaccination programs in every country by 2030 [49]. According to the estimates, 27 million children in India are likely to miss the dose of diphtheria, tetanus and pertussis vaccines [50]. To meet these targets and to achieve universal immunization, a continuous effort is must with evidence-informed strategies addressing the new challenges in the vaccination drive and strengthening the routine vaccination programs across every country in the world [51, 52].

## Supporting information

S1 Fig Spatial distribution of Standardized Incidence Ratio (SIR) of the study events across 640 districts of India, NFHS-4, 2015–16. (PDF)

S1 Table Description of reporting by vaccination dose, National Family Health Survey (NFHS), India, 2015–16. (PDF)

S2 Table Description of the predictors included in the study, National Family Health Survey (NFHS), India, 2015–16. (PDF)

S3 Table Generalized variance-inflation factor for predictors employed in selected outcomes of hepatitis-B among children aged 12–59 months, National Family Health Survey (NFHS-4), India, 2015–16. (PDF)

S4 Table Distribution of children aged 12–59 months receiving different doses of hepatitis-B vaccination, National Family Health Survey (NFHS), India, 2015–16. (PDF)

S5 Table Description of the study population (children-aged 12–59 months), National Family Health Survey (NFHS), India, 2015–16. (PDF)

S6 Table State* level prevalence (per 100 children) of missing different doses of Hepatitis-B among children aged 12–59 months, National Family Health Survey (NFHS), India, 2015–16. (PDF)

S7 Table State* level dropout rates of different doses of Hepatitis-B among children aged 12–59 months, National Family Health Survey (NFHS), India, 2015–16. (PDF)

S8 Table Spatial model output and diagnostic results of missing different doses of Hepatitis-B among children aged 12–59 months, National Family Health Survey (NFHS), India, 2015–16. (PDF)

S9 Table Spatial model output and diagnostic results of dropout of different doses of Hepatitis-B among children aged 12–59 months, National Family Health Survey (NFHS), India, 2015–16. (PDF)

S10 Table State wise identification of districts with higher posterior median risk (PMR) associated with the missing different doses of hepatitis B, National Family Health Survey (NFHS-4), 2015–16. (PDF)

S11 Table State wise identification of districts with higher posterior median risk (PMR) associated with the drop out of different doses of hepatitis B, National Family Health Survey (NFHS-4), 2015–16. (PDF)
