# Supplementary material for: Population and sub-national (district) level diversity in missed and dropout of different doses of hepatitis-B vaccine among Indian children aged 12–59 months
Source: PLOS Glob Public Health. 2022 May 17;2(5):e0000243. doi: 10.1371/journal.pgph.0000243 (PMC10021217; doi:10.1371/journal.pgph.0000243)
Supplement: S1 Fig — (PDF) [file pgph.0000243.s001.pdf]

S1 Fig. Spatial distribution of Standardized Incidence Ratio (SIR) of the study events across 640 districts of India, NFHS-4, 2015-16

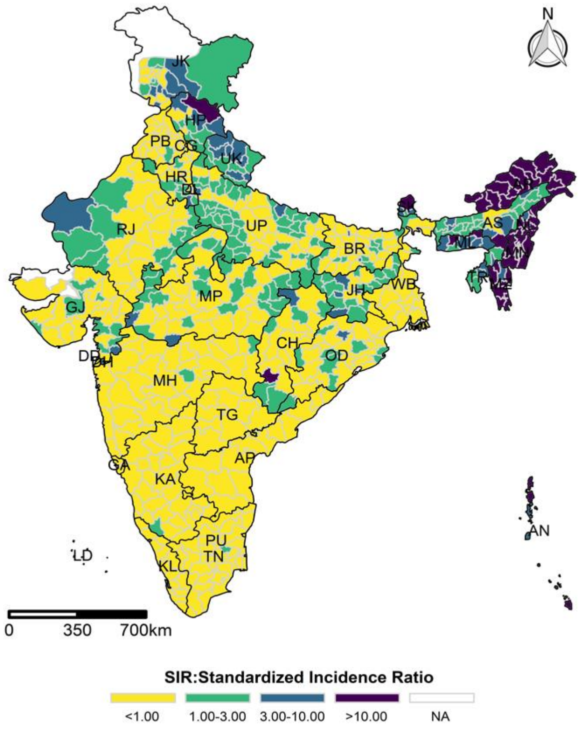

A: SIR- Missed birth dose

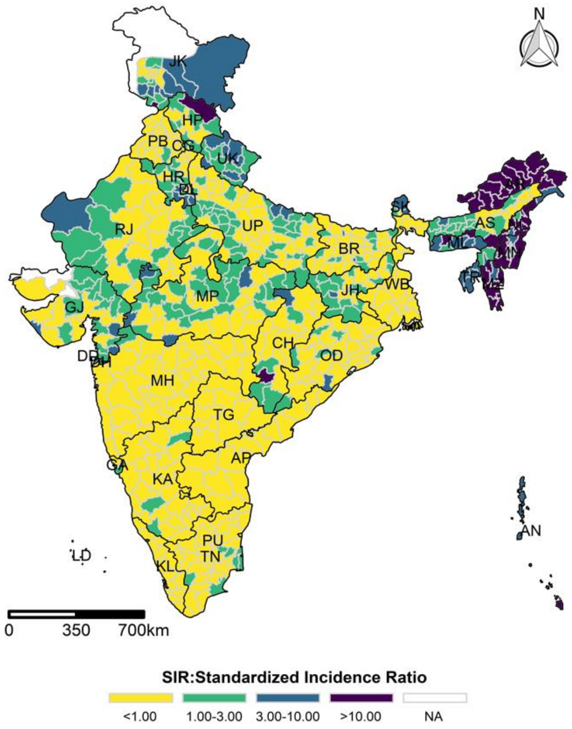

B: SIR- Missed first dose

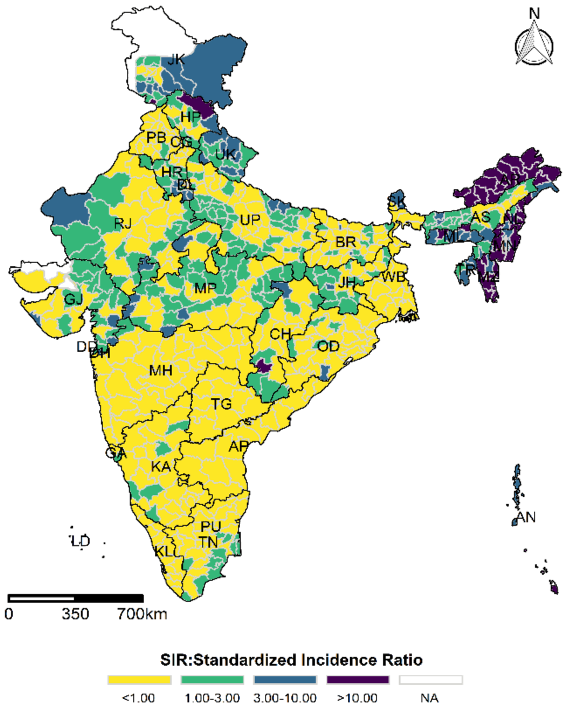

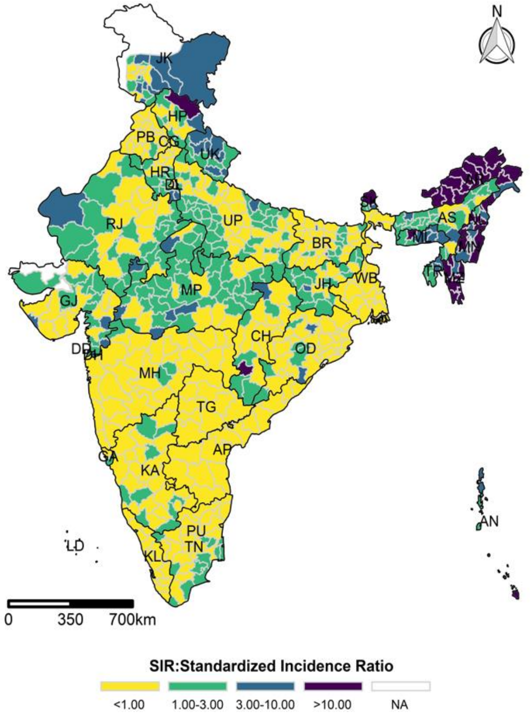

C: SIR- Missed second dose

D: SIR- Missed third dose

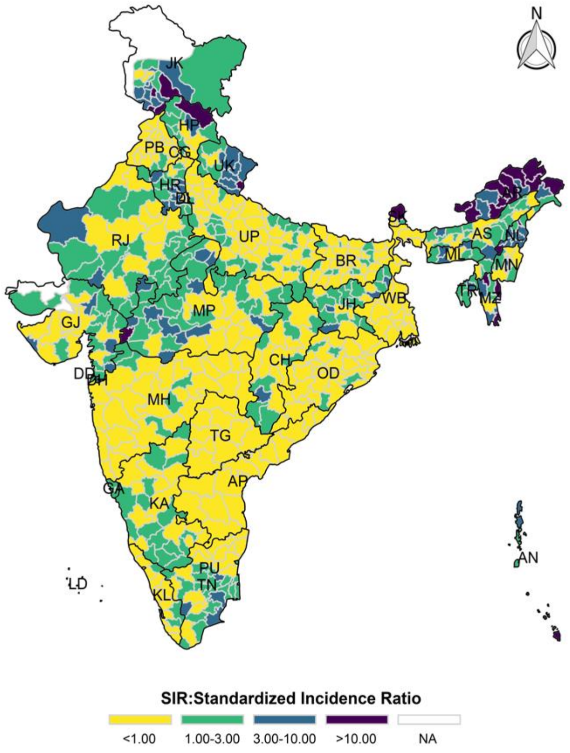

E: SIR- Dropout (0-1)

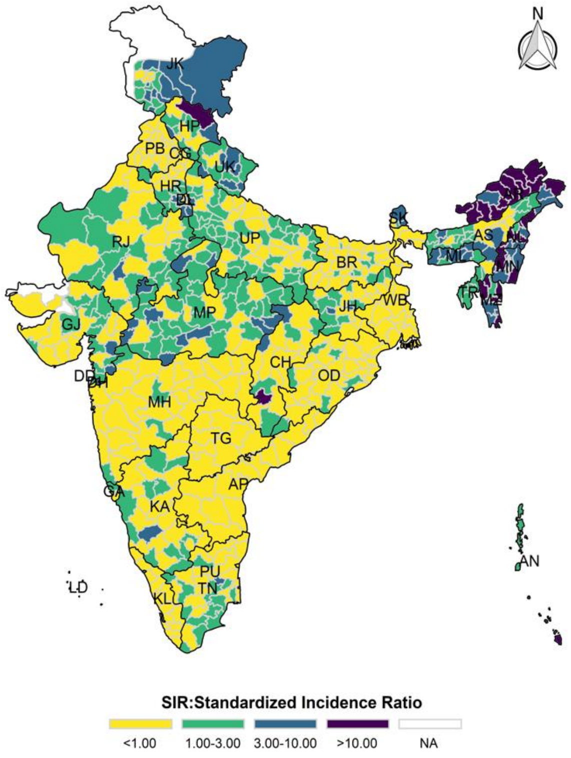

F: SIR- Dropout (1-2)

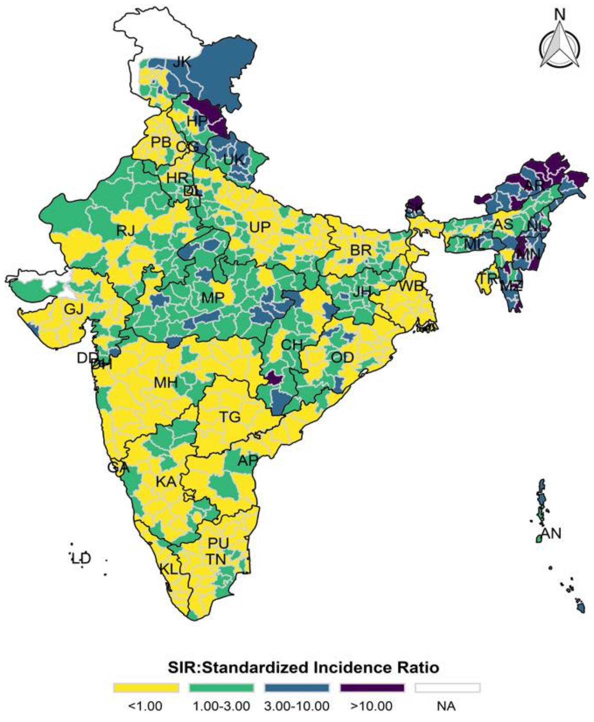

G: SIR- Dropout (2-3)
