# Supplementary material for: Population and sub-national (district) level diversity in missed and dropout of different doses of hepatitis-B vaccine among Indian children aged 12–59 months
Source: PLOS Glob Public Health. 2022 May 17;2(5):e0000243. doi: 10.1371/journal.pgph.0000243 (PMC10021217; doi:10.1371/journal.pgph.0000243)
Supplement: S1 Table — (PDF) [file pgph.0000243.s002.pdf]

S1 Table. Description of reporting by vaccination dose, National Family Health Survey (NFHS), India, 2015-16

Child's Age (In months)	Not Vaccinated	Vaccination Date on Card	Reported by mother	Vaccination Marked on Card	Don't know	Total
**Outcome 1: Birth Dose**						
12-23	33.17	38.12	22.19	5.35	1.18	100.00
24-35	34.91	31.68	27.22	4.73	1.46	100.00
36-47	37.82	24.33	31.65	4.35	1.85	100.00
48-59	40.38	19.76	34.28	3.53	2.06	100.00
Total	37.54	30.2	26.29	4.46	1.5	100.00
**Outcome 2: First Dose**						
12-23	16.76	53.94	25.25	2.88	1.18	100.00
24-35	17.14	46.88	31.92	2.59	1.46	100.00
36-47	19.85	37.25	38.42	2.63	1.85	100.00
48-59	23.21	29.3	42.91	2.53	2.06	100.00
Total	23.51	41.45	30.73	2.8	1.5	100.00
**Outcome 3: Second Dose**						
12-23	21.68	52.91	21.39	2.85	1.18	100.00
24-35	22.51	45.78	27.54	2.71	1.46	100.00
36-47	25.33	36.45	33.66	2.71	1.85	100.00
48-59	28.99	28.25	37.95	2.75	2.06	100.00
Total	29.72	39.54	26.34	2.89	1.5	100.00
**Outcome 4: Third Dose**						
12-23	35.28	50.44	10	3.1	1.18	100.00
24-35	38.77	43.94	12.81	3.03	1.46	100.00
36-47	44.25	34.75	16.11	3.05	1.85	100.00
48-59	50.31	26.31	18.11	3.21	2.06	100.00
Total	46.3	36.63	12.39	3.17	1.5	100.00
