# Supplementary material for: Population and sub-national (district) level diversity in missed and dropout of different doses of hepatitis-B vaccine among Indian children aged 12–59 months
Source: PLOS Glob Public Health. 2022 May 17;2(5):e0000243. doi: 10.1371/journal.pgph.0000243 (PMC10021217; doi:10.1371/journal.pgph.0000243)
Supplement: S2 Table — (PDF) [file pgph.0000243.s003.pdf]

S2 Table. Description of the predictors included in the study, National Family Health Survey (NFHS), India, 2015-16

Predictors	Categories
Child's age (in months)	12-23; 24-35; 36-47; 48-59
Birth order	1; 2-3; 4-5; 6 or more
Sex of the child	Male; Female
Mother's education	No education; Primary or less; Secondary or less; Higher Education
Social group	Scheduled Castes (SC); Scheduled Tribes (ST); Non-Scheduled Castes/Tribes
Religion	Hindu; Muslim; Christian; Others
Wealth quintiles	Poorest; Poorer; Middle; Richer; Richest
Place of Residence	Urban; Rural
Place of delivery	Home; Institutional
Region	North (Haryana, Himachal Pradesh, Jammu and Kashmir, Punjab, Rajasthan, Chandigarh, and Delhi)
	Central (Chhattisgarh, Madhya Pradesh, Uttrakhand, Uttar Pradesh)
	Eastern (Bihar, Jharkhand, Odisha, West Bengal)
	North-eastern (Assam, Arunachal Pradesh, Manipur, Mizoram, Meghalaya, Mizoram, Nagaland, Sikkim, and Tripura)
	Western (Goa, Gujarat, Maharashtra, Dadra and Nagar Haveli and Daman and Diu), and
	Southern (Andhra Pradesh, Karnataka, Kerala, Tamil Nadu, Telangana, Andaman and Nicobar Islands, Lakshadweep, and Puducherry)
