# Supplementary material for: Population and sub-national (district) level diversity in missed and dropout of different doses of hepatitis-B vaccine among Indian children aged 12–59 months
Source: PLOS Glob Public Health. 2022 May 17;2(5):e0000243. doi: 10.1371/journal.pgph.0000243 (PMC10021217; doi:10.1371/journal.pgph.0000243)
Supplement: S3 Table — (PDF) [file pgph.0000243.s004.pdf]

S3 Table. Generalized variance-inflation factor for predictors employed in selected outcomes of hepatitis-B among children aged 12-59 months, National Family Health Survey (NFHS-4), India, 2015-16.

Predictors	Birth Dose		First Dose		Second Dose		Third Dose		First Dose Dropout		Second Dose Dropout		Third Dose Dropout	
	GVIF	AGVIF	GVIF	AGVIF	GVIF	AGVIF	GVIF	AGVIF	GVIF	AGVIF	GVIF	AGVIF	GVIF	AGVIF
**Child Age (in months**														
0-5	NA	NA	NA	NA	NA	NA	NA	NA	NA	NA	NA	NA	NA	NA
6-11	1.73	1.31	1.63	1.28	1.86	1.36	2.49	1.58	1.26	1.12	1.42	1.19	1.88	1.37
12-23	2.23	1.49	1.91	1.38	2.28	1.51	3.45	1.86	1.26	1.12	1.56	1.25	2.50	1.58
24-35	2.24	1.50	1.92	1.38	2.29	1.51	3.48	1.86	1.21	1.10	1.56	1.25	2.67	1.63
36-47	2.31	1.52	2.03	1.43	2.42	1.56	3.61	1.90	1.23	1.11	1.59	1.26	2.85	1.69
48-59	2.28	1.51	2.08	1.44	2.46	1.57	3.53	1.88	1.24	1.11	1.58	1.26	2.89	1.70
**Birth Order**														
1	NA	NA	NA	NA	NA	NA	NA	NA	NA	NA	NA	NA	NA	NA
2 to 3	1.26	1.12	1.31	1.14	1.29	1.14	1.25	1.12	1.21	1.10	1.28	1.13	1.25	1.12
4 to 5	1.31	1.15	1.36	1.17	1.35	1.16	1.31	1.14	1.27	1.13	1.34	1.16	1.32	1.15
6+	1.16	1.08	1.20	1.10	1.19	1.09	1.16	1.08	1.13	1.07	1.18	1.09	1.16	1.08
**Sex**														
Male	NA	NA	NA	NA	NA	NA	NA	NA	NA	NA	NA	NA	NA	NA
Female	1.00	1.00	1.00	1.00	1.00	1.00	1.00	1.00	1.00	1.00	1.00	1.00	1.00	1.00
**Mother Education**														
No	NA	NA	NA	NA	NA	NA	NA	NA	NA	NA	NA	NA	NA	NA
Primary or less	1.31	1.15	1.28	1.13	1.29	1.14	1.33	1.15	1.35	1.16	1.32	1.15	1.32	1.15
Secondary or less	1.89	1.37	1.81	1.35	1.84	1.36	1.92	1.38	1.95	1.40	1.90	1.38	1.93	1.39
Higher	1.68	1.30	1.57	1.25	1.61	1.27	1.73	1.31	1.75	1.32	1.71	1.31	1.80	1.34
**Caste**														
Scheduled Castes	NA	NA	NA	NA	NA	NA	NA	NA	NA	NA	NA	NA	NA	NA
Scheduled Tribes	2.07	1.44	2.24	1.50	2.19	1.48	2.12	1.46	1.88	1.37	2.06	1.44	1.95	1.40
Others	1.79	1.34	1.90	1.38	1.87	1.37	1.81	1.34	1.73	1.31	1.79	1.34	1.75	1.32
**Religion**														
Hindu	NA	NA	NA	NA	NA	NA	NA	NA	NA	NA	NA	NA	NA	NA
Muslim	1.16	1.08	1.19	1.09	1.18	1.09	1.16	1.08	1.14	1.07	1.17	1.08	1.15	1.07

Christian	1.85	1.36	2.11	1.45	2.05	1.43	1.97	1.40	1.67	1.29	1.93	1.39	1.77	1.33
Others	1.10	1.05	1.10	1.05	1.10	1.05	1.10	1.05	1.07	1.04	1.09	1.04	1.08	1.04
**Wealth Index**														
Poorest	NA	NA	NA	NA	NA	NA	NA	NA	NA	NA	NA	NA	NA	NA
Poorer	1.61	1.27	1.56	1.25	1.58	1.26	1.65	1.28	1.70	1.30	1.60	1.27	1.61	1.27
Middle	1.82	1.35	1.73	1.32	1.77	1.33	1.86	1.36	1.91	1.38	1.80	1.34	1.82	1.35
Richer	2.03	1.42	1.92	1.38	1.97	1.40	2.10	1.45	2.15	1.47	2.06	1.44	2.10	1.45
Richest	2.33	1.53	2.14	1.46	2.23	1.49	2.45	1.57	2.46	1.57	2.35	1.53	2.50	1.58
**Residence**														
Urban	NA	NA	NA	NA	NA	NA	NA	NA	NA	NA	NA	NA	NA	NA
Rural	1.32	1.15	1.32	1.15	1.32	1.15	1.33	1.15	1.31	1.14	1.33	1.15	1.34	1.16
**Place of Delivery**														
Home	NA	NA	NA	NA	NA	NA	NA	NA	NA	NA	NA	NA	NA	NA
Institutional	1.15	1.07	1.21	1.10	1.20	1.10	1.19	1.09	1.13	1.06	1.21	1.10	1.19	1.09
**Regions**														
North	NA	NA	NA	NA	NA	NA	NA	NA	NA	NA	NA	NA	NA	NA
Central	2.04	1.43	2.04	1.43	2.03	1.42	1.96	1.40	1.80	1.34	2.09	1.45	2.10	1.45
East	1.99	1.41	1.93	1.39	1.91	1.38	1.94	1.39	1.74	1.32	1.74	1.32	1.95	1.40
North-East	2.21	1.49	2.47	1.57	2.41	1.55	2.30	1.52	1.89	1.37	2.29	1.51	2.10	1.45
West	1.36	1.17	1.37	1.17	1.36	1.16	1.33	1.15	1.29	1.14	1.36	1.17	1.39	1.18
South	1.39	1.18	1.38	1.18	1.41	1.19	1.46	1.21	1.42	1.19	1.51	1.23	1.51	1.23

GVIF: Generalized Variance Inflation Factor, AGVIF: Adjusted Generalized Variance Inflation Factor, df: Degrees of Freedom
AGVIF=GVIF^(1/(2*df))
