# Supplementary material for: Population and sub-national (district) level diversity in missed and dropout of different doses of hepatitis-B vaccine among Indian children aged 12–59 months
Source: PLOS Glob Public Health. 2022 May 17;2(5):e0000243. doi: 10.1371/journal.pgph.0000243 (PMC10021217; doi:10.1371/journal.pgph.0000243)
Supplement: S4 Table — (PDF) [file pgph.0000243.s005.pdf]

S4 Table. Distribution of children aged 12-59 months receiving different doses of hepatitis-B vaccination, National Family Health Survey (NFHS), India, 2015-16

Hepatitis Dose Received	Category	Frequency	Percent
**Hepatitis-b 0**	No	77,948	39.6
	Reported on vaccination card	63,291	32.2
	Reported by mother	51,730	26.3
	Don't know	3,685	1.9
**Hepatitis-b 1**	No	41,607	21.2
	Reported on vaccination card	85,363	43.4
	Reported by mother	65,999	33.6
	Don't know	3,685	1.9
**Hepatitis-b 2**	No	51,672	26.3
	Reported on vaccination card	83,531	42.5
	Reported by mother	57,766	29.4
	Don't know	3,685	1.9
**Hepatitis-b 3**	No	83,888	42.7
	Reported on vaccination card	80,188	38.2
	Reported by mother	28,893	14.7
	Don't know	3,685	1.9
**Total**		1,96,654	100.0
