# Supplementary material for: Population and sub-national (district) level diversity in missed and dropout of different doses of hepatitis-B vaccine among Indian children aged 12–59 months
Source: PLOS Glob Public Health. 2022 May 17;2(5):e0000243. doi: 10.1371/journal.pgph.0000243 (PMC10021217; doi:10.1371/journal.pgph.0000243)
Supplement: S5 Table — (PDF) [file pgph.0000243.s006.pdf]

S5 Table. Description of the study population (children-aged 12-59 months), National Family Health Survey (NFHS), India, 2015-16

Background characteristics	Frequency	Percentage
**Child's age (in months)**		
12-23	48,928	24.9
24-35	48,517	24.7
36-47	50,697	25.8
48-59	48,512	24.7
**Birth order**		
1	72,500	36.9
2_3	92,298	46.9
4-5	23,754	12.1
6+	8,102	4.1
**Sex of the child**		
Male	1,02,036	51.9
Female	94,618	48.1
**Mother's education**		
No education	62,151	31.6
Primary or less	28,978	14.7
Secondary or less	87,696	44.6
Higher Education	17,829	9.1
**Social group**		
Scheduled Castes (SC)	36,789	18.7
Scheduled Tribes (ST)	39,356	20.0
Non-Scheduled Castes/Tribes	1,20,509	61.3
**Religion**		
Hindu	1,41,696	72.1
Muslim	31,026	15.8
Christian	16,101	8.2
Others	7,831	4.0
**Wealth quintiles[£]**		
Poorest	51,477	26.2
Poorer	46,117	23.5
Middle	39,234	20.0
Richer	32,983	16.8
Richest	26,843	13.7
**Place of Residence**		
Urban	47,509	24.2
Rural	1,49,145	75.8
**Place of delivery**		
Home	49,278	25.1
Institutional	1,47,376	74.9
**Region**		
North	37,139	18.9
Central	55,902	28.4
East	40,969	20.8
North East	28,706	14.6
West	14,072	7.2
South	19,866	10.1
**Total**	**1,96,654**	**100.0**

£ wealth quintiles denote five different economic classes of India. [1] Children who received the birth dose; [2] Children who received the first dose; [3] Children who received the second dose
