# Supplementary material for: Population and sub-national (district) level diversity in missed and dropout of different doses of hepatitis-B vaccine among Indian children aged 12–59 months
Source: PLOS Glob Public Health. 2022 May 17;2(5):e0000243. doi: 10.1371/journal.pgph.0000243 (PMC10021217; doi:10.1371/journal.pgph.0000243)
Supplement: S6 Table — (PDF) [file pgph.0000243.s007.pdf]

S6 Table. State* level prevalence (per 100 children) of missing different doses of Hepatitis-B among children aged 12-59 months, National Family Health Survey (NFHS), India, 2015-16

States	Birth dose	First dose	Second dose	Third dose	Number of Children
Andaman and Nicobar	41.39	19.59	20.60	25.99	503
Andhra Pradesh	15.65	8.63	15.31	44.77	2,360
Arunachal Pradesh	75.96	46.44	53.22	64.97	3,917
Assam	58.21	27.89	32.98	47.57	7,909
Bihar	42.23	24.79	28.74	46.18	19,045
Chandigarh	17.95	3.26	3.89	11.83	154
Chhattisgarh	22.96	8.76	11.59	30.10	6,615
Dadra &Nagar Haveli	41.90	25.69	31.26	50.65	249
Daman and Diu	35.22	26.53	35.29	54.31	310
Delhi	25.45	12.33	16.97	33.97	1,220
Goa	10.16	9.05	11.74	20.61	337
Gujarat	45.79	38.89	46.73	67.99	5,885
Haryana	34.90	28.55	34.48	47.12	5,991
Himachal Pradesh	35.36	13.26	17.60	32.02	2,221
Jammu &Kashmir	18.17	17.44	21.95	31.01	6,302
Jharkhand	45.95	19.65	23.51	40.15	9,268
Karnataka	20.83	14.86	21.70	41.22	5,863
Kerala	23.63	6.71	8.34	16.95	1,995
Lakshadweep	14.50	2.84	3.65	11.73	233
Madhya Pradesh	30.74	21.78	28.60	51.58	18,461
Maharashtra	31.70	18.56	23.98	45.53	7,291
Manipur	77.14	25.66	30.29	41.23	4,360
Meghalaya	69.70	34.46	38.15	48.45	3,451
Mizoram	66.49	35.91	38.95	46.75	3,794
Nagaland	75.81	44.56	50.44	59.36	3,485
Odisha	28.83	12.64	14.67	24.91	8,490
Puducherry	8.94	5.95	7.73	17.34	842
Punjab	6.03	4.03	4.95	8.75	4,052
Rajasthan	35.69	23.67	30.14	51.18	12,773
Sikkim	25.23	6.83	8.36	17.17	740
Tamil Nadu	20.25	16.06	22.22	34.96	6,207
Telangana	17.40	10.40	18.98	42.15	1,863
Tripura	72.21	54.27	59.96	65.59	1,050
Uttar Pradesh	58.05	30.14	36.98	55.09	30,826
Uttarakhand	45.56	25.48	30.74	49.40	4,426
West Bengal	51.89	8.27	10.82	20.48	4,166
**Total**	**38.21**	**20.88**	**26.26**	**43.79**	**1,96,654**

*States in India are the first administrative units
