# Supplementary material for: Population and sub-national (district) level diversity in missed and dropout of different doses of hepatitis-B vaccine among Indian children aged 12–59 months
Source: PLOS Glob Public Health. 2022 May 17;2(5):e0000243. doi: 10.1371/journal.pgph.0000243 (PMC10021217; doi:10.1371/journal.pgph.0000243)
Supplement: S7 Table — (PDF) [file pgph.0000243.s008.pdf]

S7 Table. State* level dropout rates of different doses of Hepatitis-B among children aged 12-59 months, National Family Health Survey (NFHS), India, 2015-16

States	First dose dropout	No of children[1]	Second dose dropout	No of children[2]	Third dose dropout	No of children[3]
Andaman and Nicobar	1.71	245	2.15	344	7.27	337
Andhra Pradesh	4.10	1,982	7.53	2,157	34.93	1,999
Arunachal Pradesh	10.65	960	15.95	1,944	25.96	1,687
Assam	6.42	3,411	7.95	5,768	22.44	5,368
Bihar	5.03	11,448	5.90	14,737	25.09	14,040
Chandigarh	1.56	126	0.65	148	8.26	147
Chhattisgarh	3.05	5,070	3.25	5,981	21.12	5,791
Dadra &Nagar Haveli	4.98	144	9.08	185	29.35	170
Daman and Diu	7.10	218	12.54	246	29.39	226
Delhi	4.98	874	5.50	1,038	20.82	981
Goa	4.12	295	2.96	302	10.04	291
Gujarat	11.77	3,190	13.33	3,541	40.26	3,109
Haryana	9.59	3,851	8.66	4,231	19.50	3,913
Himachal Pradesh	4.16	1,378	5.39	1,906	17.62	1,820
Jammu &Kashmir	7.76	5,093	5.68	5,182	12.34	4,919
Jharkhand	7.96	4,976	5.34	7,484	22.29	7,129
Karnataka	4.89	4,756	8.51	4,989	25.39	4,628
Kerala	2.47	1,565	2.21	1,864	9.72	1,830
Lakshadweep	0.93	197	1.24	226	8.80	224
Madhya Pradesh	6.46	12,613	9.03	14,237	32.43	12,905
Maharashtra	4.81	4,989	7.20	6,067	28.75	5,728
Manipur	4.96	940	6.72	3,198	16.07	2,979
Meghalaya	6.01	1,134	6.39	2,321	17.18	2,208
Mizoram	4.17	1,187	4.99	2,447	12.99	2,310
Nagaland	9.18	847	11.30	1,945	18.65	1,747
Odisha	1.75	6,224	2.64	7,528	12.22	7,366
Puducherry	2.21	754	1.94	799	10.60	783
Punjab	1.20	3,813	0.98	3,897	4.01	3,864
Rajasthan	6.68	8,297	8.71	9,851	30.33	9,049
Sikkim	0.32	562	1.89	694	9.62	685
Tamil Nadu	7.52	4,902	7.73	5,172	17.06	4,760
Telangana	3.43	1,508	9.79	1,645	28.63	1,504
Tripura	15.85	271	13.57	472	15.28	416
Uttar Pradesh	7.44	12,844	10.48	21,702	29.44	19,623
Uttarakhand	7.59	2,411	7.65	3,312	27.31	3,078
West Bengal	2.39	1,946	3.07	3,802	11.04	3,683
**Total**	**5.61**	**1,15,021**	**7.26**	**1,51,362**	**24.18**	**1,41,297**

[1] Children who received the birth dose
[2] Children who received the first dose
[3] Children who received the second dose
*States in India are the first administrative units
