# Supplementary material for: Population and sub-national (district) level diversity in missed and dropout of different doses of hepatitis-B vaccine among Indian children aged 12–59 months
Source: PLOS Glob Public Health. 2022 May 17;2(5):e0000243. doi: 10.1371/journal.pgph.0000243 (PMC10021217; doi:10.1371/journal.pgph.0000243)
Supplement: S8 Table — (PDF) [file pgph.0000243.s009.pdf]

S8 Table. Spatial model output and diagnostic results of missing different doses of Hepatitis-B among children aged 12-59 months, National Family Health Survey (NFHS), India, 2015-16

**Spatial and diagnostic Results**	**Birth dose**	**First Dose**	**Second Dose**	**Third Dose**
Moran's I of residuals from the fitted Poisson' model	0.406 ***	0.419 ***	0.413 ***	0.398 ***
**DIC Value**	**Birth dose**	**First Dose**	**Second Dose**	**Third Dose**
Bayesian CARleroux Model	**5302.74**	**4886.29**	5049.23	5423.25
Bayesian CARBym Model	5304.27	4888.57	**5047.36**	**5418.96**
Gelman-Rubin Statistic (Convergence Check)	All the scale reduction factors are <1.1	All the scale reduction factors are <1.1	All the scale reduction factors are <1.1	All the scale reduction factors are <1.1
**Fitted Model Output**	**Posterior Median (95% Credible Intervals)**	**Posterior Median (95% Credible Intervals)**	**Posterior Median (95% Credible Intervals)**	**Posterior Median (95% Credible Intervals)**
Intercept	-0.943 (-1.30; -0.573)	-1.083 (-1.488; -0.677)	-0.966 (-1.362; -0.569)	-0.665 (-1.023; -0.315)
No Education (%)	0.002 (-0.006; 0.010)	0.007 (-0.001; 0.016)	0.008 (0.000; 0.016)	0.007 (0.000; 0.015)
Home Delivery (%)	0.025 (0.018 ;0.033)	0.025 (0.017; 0.034)	0.025 (0.017; 0.033)	0.020 (0.013; 0.027)
Poor (%)	-0.004 (-0.012 ;0.002)	-0.004 (-0.013; 0.004)	-0.007 (-0.014; 0.001)	-0.007 (-0.014;0.000)
Rural (%)	0.008 (0.003; 0.013)	0.008 (0.002; 0.014)	0.008 (0.003; 0.013)	0.00785 (0.003; 0.013)
Scheduled Castes (%)	-0.006 (-0.014; 0.003)	-0.008 (-0.017; 0.001)	-0.007 (-0.016; 0.002)	-0.009 (-0.017;0.001)
Non-Hindu (%)	0.003 (-0.002; 0.007)	0.003 (-0.001; 0.008)	0.002 (-0.002; 0.006)	0.002 (-0.002; 0.007)

Note. *p < 0.05, **p < 0.01, ***p < 0.001
