# Supplementary material for: Population and sub-national (district) level diversity in missed and dropout of different doses of hepatitis-B vaccine among Indian children aged 12–59 months
Source: PLOS Glob Public Health. 2022 May 17;2(5):e0000243. doi: 10.1371/journal.pgph.0000243 (PMC10021217; doi:10.1371/journal.pgph.0000243)
Supplement: S9 Table — (PDF) [file pgph.0000243.s010.pdf]

S9 Table. Spatial model output and diagnostic results of dropout of different doses of Hepatitis-B among children aged 12-59 months, National Family Health Survey (NFHS), India, 2015-16

**Spatial and diagnostic Results**	**Dropout (0-1)**	**Dropout (1-2)**	**Dropout (2-3)**
Moran's I of residuals from the fitted Poisson' model	0.342 ***	0.320 ***	0.337 ***
**DIC Value**	**Dropout (0-1)**	**Dropout (1-2)**	**Dropout (2-3)**
Bayesian CARleroux Model	3612.38	3963.15	**4784**
Bayesian CARBym Model	**3612.27**	**3961.33**	4784.08
Gelman-Rubin Statistic (Convergence Check)	All the scale reduction factors are <1.1	All the scale reduction factors are <1.1	All the scale reduction factors are <1.1
**Fitted Model Output**	**Posterior Median (95% Credible Intervals)**	**Posterior Median (95% Credible Intervals)**	**Posterior Median (95% Credible Intervals)**
Intercept	-0.495 (-0.930; -0.055)	-0.53763 (-0.940; -0.144)	-0.268 (-0.611; 0.071)
No Education (%)	0.007 (-0.002; 0.016)	0.008 (0.000; 0.017)	0.003 (-0.004; 0.010)
Home Delivery (%)	0.012 (0.004; 0.021)	0.021 (0.013; 0.029)	0.011 (0.004; 0.018)
Poor (%)	-0.011 (-0.020; -0.003)	-0.011 (-0.019; -0.004)	-0.003 (-0.010; 0.004)
Rural (%)	0.012 (0.006; 0.018)	0.008 (0.002; 0.013)	0.006 (0.001; 0.011)
Scheduled Castes (%)	-0.012 (-0.023; -0.002)	-0.006 (-0.016; 0.003)	-0.013 (-0.021; -0.005)
Non-Hindu (%)	-0.003 (-0.009; 0.002)	-0.001 (-0.005; 0.004)	0.001 (-0.002; 0.006)

Note. *p < 0.05, **p < 0.01, ***p < 0.001
