# Supplementary material for: Population and sub-national (district) level diversity in missed and dropout of different doses of hepatitis-B vaccine among Indian children aged 12–59 months
Source: PLOS Glob Public Health. 2022 May 17;2(5):e0000243. doi: 10.1371/journal.pgph.0000243 (PMC10021217; doi:10.1371/journal.pgph.0000243)
Supplement: S10 Table — (PDF) [file pgph.0000243.s011.pdf]

S10 Table. State wise identification of districts with higher posterior median risk (PMR) associated with the missing different doses of hepatitis B, National Family Health Survey (NFHS-4), 2015-16

States/UTs	No. of Districts	Birth dose		First dose		Second dose		Third dose	
		No. of Districts at Risk	Prop of Districts at Risk	No. of Districts at Risk	Prop of Districts at Risk	No. of Districts at Risk	Prop of Districts at Risk	No. of Districts at Risk	Prop of Districts at Risk
Andaman & Nicobar Islands	03	03	100.0	3	100.0	3	100.0	3	100.0
Andhra Pradesh	22	00	0.0	0	0.0	0	0.0	0	0.0
Arunachal Pradesh	16	16	100.0	16	100.0	16	100.0	16	100.0
Assam	27	25	92.6	20	74.1	21	77.8	22	81.5
Bihar	38	12	31.6	11	28.9	11	28.9	14	36.8
Chandigarh	01	00	0.0	0	0.0	0	0.0	0	0.0
Chhattisgarh	18	07	38.9	4	22.2	7	38.9	8	44.4
Dadra & Nagar Haveli	01	01	100.0	1	100.0	1	100.0	1	100.0
Daman & Diu	02	02	100.0	2	100.0	2	100.0	2	100.0
Goa	02	00	0.0	1	50.0	1	50.0	1	50.0
Gujarat	28	08	28.6	17	60.7	18	64.3	15	53.6
Haryana	21	09	42.9	15	71.4	15	71.4	13	61.9
Himachal Pradesh	12	10	83.3	8	66.7	8	66.7	8	66.7
Jammu & Kashmir	23	11	47.8	16	69.6	17	73.9	15	65.2
Jharkhand	24	19	79.2	16	66.7	16	66.7	18	75.0
Karnataka	30	01	3.3	3	10.0	5	16.7	9	30.0
Kerala	14	00	0.0	0	0.0	0	0.0	0	0.0
Lakshadweep	01	01	100.0	1	100.0	1	100.0	1	100.0
Madhya Pradesh	50	24	48.0	29	58.0	32	64.0	40	80.0
Maharashtra	35	02	5.7	1	2.9	1	2.9	3	8.6
Manipur	09	09	100.0	9	100.0	9	100.0	9	100.0
Meghalaya	07	07	100.0	7	100.0	7	100.0	7	100.0
Mizoram	08	08	100.0	8	100.0	8	100.0	8	100.0
Nagaland	11	11	100.0	11	100.0	11	100.0	11	100.0
NCT Of Delhi	09	01	11.1	2	22.2	2	22.2	3	33.3
Odisha	30	10	33.3	5	16.7	5	16.7	7	23.3
Puducherry	03	02	66.7	2	66.7	2	66.7	2	66.7
Punjab	20	01	5.0	1	5.0	1	5.0	1	5.0
Rajasthan	33	12	36.4	14	42.4	15	45.5	21	63.6
Sikkim	04	04	100.0	3	75.0	2	50.0	4	100.0
Tamil Nadu	32	01	3.1	6	18.8	10	31.3	11	34.4
Tripura	04	04	100.0	4	100.0	4	100.0	4	100.0
Uttar Pradesh	71	46	64.8	35	49.3	39	54.9	42	59.2
Uttarakhand	13	13	100.0	13	100.0	13	100.0	13	100.0
West Bengal	19	02	10.5	0	0.0	0	0.0	0	0.0
**Total Districts**	**641**	**282**	**44.0**	**284**	**44.3**	**303**	**47.3**	**332**	**51.8**
