# Supplementary material for: Population and sub-national (district) level diversity in missed and dropout of different doses of hepatitis-B vaccine among Indian children aged 12–59 months
Source: PLOS Glob Public Health. 2022 May 17;2(5):e0000243. doi: 10.1371/journal.pgph.0000243 (PMC10021217; doi:10.1371/journal.pgph.0000243)
Supplement: S11 Table — (PDF) [file pgph.0000243.s012.pdf]

**S11 Table.** State wise identification of districts with higher posterior median risk (PMR) associated with the drop out of different doses of hepatitis B, National Family Health Survey (NFHS-4), 2015-16

| States/UTs | No. of Districts | Dropout (0-1) | | Dropout (1-2) | | Dropout (2-3) | |
|---|---|---|---|---|---|---|---|
| | | No. of Districts at Risk | Proportion of Districts at Risk | No. of Districts at Risk | Proportion of Districts at Risk | No. of Districts at Risk | Proportion of Districts at Risk |
| Andaman & Nicobar Islands | 03 | 03 | 100.0 | 3 | 100.0 | 2 | 66.7 |
| Andhra Pradesh | 22 | 00 | 0.0 | 0 | 0.0 | 3 | 13.6 |
| Arunachal Pradesh | 16 | 15 | 93.8 | 16 | 100.0 | 16 | 100.0 |
| Assam | 27 | 18 | 66.7 | 22 | 81.5 | 21 | 77.8 |
| Bihar | 38 | 10 | 26.3 | 6 | 15.8 | 16 | 42.1 |
| Chandigarh | 01 | 00 | 0.0 | 0 | 0.0 | 0 | 0.0 |
| Chhattisgarh | 18 | 09 | 50.0 | 6 | 33.3 | 15 | 83.3 |
| Dadra & Nagar Haveli | 01 | 01 | 100.0 | 1 | 100.0 | 1 | 100.0 |
| Daman & Diu | 02 | 02 | 100.0 | 2 | 100.0 | 2 | 100.0 |
| Goa | 2 | 02 | 100.0 | 1 | 50.0 | 0 | 0.0 |
| Gujarat | 28 | 18 | 64.3 | 14 | 50.0 | 11 | 39.3 |
| Haryana | 21 | 18 | 85.7 | 12 | 57.1 | 11 | 52.4 |
| Himachal Pradesh | 12 | 08 | 66.7 | 8 | 66.7 | 10 | 83.3 |
| Jammu & Kashmir | 23 | 20 | 87.0 | 19 | 82.6 | 13 | 56.5 |
| Jharkhand | 24 | 19 | 79.2 | 12 | 50.0 | 20 | 83.3 |
| Karnataka | 30 | 15 | 50.0 | 10 | 33.3 | 12 | 40.0 |
| Kerala | 14 | 02 | 14.3 | 0 | 0.0 | 0 | 0.0 |
| Lakshadweep | 01 | 01 | 100.0 | 1 | 100.0 | 1 | 100.0 |
| Madhya Pradesh | 50 | 33 | 66.0 | 40 | 80.0 | 46 | 92.0 |
| Maharashtra | 35 | 04 | 11.4 | 2 | 5.7 | 5 | 14.3 |
| Manipur | 09 | 09 | 100.0 | 9 | 100.0 | 9 | 100.0 |
| Meghalaya | 07 | 06 | 85.7 | 7 | 100.0 | 7 | 100.0 |
| Mizoram | 08 | 08 | 100.0 | 8 | 100.0 | 8 | 100.0 |
| Nagaland | 11 | 11 | 100.0 | 11 | 100.0 | 11 | 100.0 |
| NCT Of Delhi | 09 | 02 | 22.2 | 0 | 0.0 | 2 | 22.2 |
| Odisha | 30 | 02 | 6.7 | 3 | 10.0 | 11 | 36.7 |
| Puducherry | 03 | 02 | 66.7 | 2 | 66.7 | 2 | 66.7 |
| Punjab | 20 | 02 | 10.0 | 1 | 5.0 | 1 | 5.0 |
| Rajasthan | 33 | 21 | 63.6 | 20 | 60.6 | 25 | 75.8 |
| Sikkim | 04 | 01 | 25.0 | 3 | 75.0 | 4 | 100.0 |
| Tamil Nadu | 32 | 17 | 53.1 | 14 | 43.8 | 7 | 21.9 |
| Tripura | 04 | 04 | 100.0 | 4 | 100.0 | 1 | 25.0 |
| Uttar Pradesh | 71 | 22 | 31.0 | 46 | 64.8 | 33 | 46.5 |
| Uttarakhand | 13 | 12 | 92.3 | 12 | 92.3 | 13 | 100.0 |
| West Bengal | 19 | 00 | 0.0 | 0 | 0.0 | 0 | 0.0 |
| **Total Districts** | **641** | **317** | **49.5** | **315** | **49.1** | **339** | **52.9** |
